# The role of astrocytes in the temporoammonic pathway: masticatory behavior as a neuroprotective strategy against age-related cognitive decline

**DOI:** 10.1186/s12993-026-00358-6

**Published:** 2026-07-28

**Authors:** Micaele Maria Lopes Castro, Fabio Leite do Amaral, Fabíola de Carvalho Chaves de Siqueira Mendes, Daniel Clive Anthony, Dora Maria Tuna de Oliveira Brites, Cristovam Wanderley Picanço Diniz, Marcia Consentino Kronka Sosthenes

**Affiliations:** 1 Laboratório de Investigações em Neurodegeneração e Infecção, Instituto de Ciências Biológicas, Hospital Universitário João de Barros Barreto, Universidade Federal do Pará, Rua Mundurucus 4487, CEP66073005, Belém, PA 66073-005 Brazil; 2 Curso de Medicina, Centro Universitário do Estado do Pará, Belém, PA 66613-903 Brazil; 3 Grupo de Pesquisa em Neurociências Teórica e Aplicada, Programa de Pós- graduação em Ciências da Saúde, Centro Universitário do Estado do Pará, Belém, PA 66613-903 Brazil; 4 Laboratory of Experimental Neuropathology, Department of Pharmacology, University of Oxford, Oxford, OX13QT UK; 5 Research Institute for Medicines (iMed.ULisboa), Faculty of Pharmacy, Universidade de Lisboa, Lisbon, Portugal; 6 Department of Pharmaceutical Sciences and Medicines, Faculty of Pharmacy, Universidade de Lisboa, Lisbon, Portugal

**Keywords:** Astrocytic plasticity, Temporoammonic pathway, Spatial memory, Masticatory activity, Age-related cognitive decline, CA1 hippocampus, Morris Water Maze (MWM)

## Abstract

Astrocytes undergo phenotypic changes with aging, contributing to neurodegenerative diseases and cognitive impairments in later life. The temporoammonic (TA) pathway terminates at the *stratum lacunosum-moleculare* (SLM) of the CA1 region, where astrocytic support is crucial for synaptic plasticity and information processing related to spatial learning and memory. This study tested the hypothesis that age-related morphological changes in astrocytes of the SLM affect cognitive performance and we explored whether masticatory activity modulates these changes. Young (6 months) and aged (18 months) female *Swiss albino* mice were subjected to three distinct masticatory regimens: a hard diet (HD), HD followed by a soft diet (HD/SD), or HD followed by SD and a return to HD (HD/SD/HD). Cognitive performance was assessed using the Morris Water Maze (MWM), with learning rates calculated from escape latencies throughout five days of trials. After behavioral testing, the mice were culled and immunohistochemical analysis of glial fibrillary acidic protein (GFAP) expression was performed. 3D reconstructions of astrocytes within the SLM were generated and analyzed. Hierarchical clustering identified distinct astrocyte morphotypes, revealing a significant age-related shift from high-complexity astrocytes (AST1) toward lower-complexity subtypes (AST2 and AST3). In the AST1 phenotype, aging did not significantly alter astrocytic complexity in animals maintained under normal masticatory conditions (HD group) in the dorsal region, then, AST1 may represent a more stable and aging-driven astrocytic phenotype, with limited sensitivity to masticatory modulation during advanced aging. Therefore, in AST2 of HD group, aged animals exhibited greater complexity compared to young animals, suggesting the occurrence of adaptive or compensatory astroglial remodeling during physiological aging. Behavioral analysis showed an age-dependent reduction in learning performance in the HD group, where 18 M mice exhibited a significantly lower contrast index than 6 M old mice on day 4 of the Morris Water Maze task. These findings highlight that morphological changes in astrocytes within the dorsal SLM may contribute to age-related alterations in hippocampus-dependent learning. Overall, maintaining proper mastication may be an effective approach to maintain astrocytic integrity during aging and preserve hippocampus-dependent cognitive function, particularly in older individuals.

**Supplementary Information:**

The online version contains supplementary material available at https://doi.org/10.1186/s12993-026-00358-6.

## Introduction

The interaction between masticatory activity, aging, and brain plasticity is gaining attention as studies increasingly suggest that oral functions such as chewing play a critical role in preserving cognitive health. Research has shown that reduced masticatory activity is associated with cognitive decline and neurodegenerative conditions, especially in the elderly population [1–3]. The hippocampus, a brain region essential for learning and memory, is particularly prone to age-related changes [4–6]. Within the hippocampus, the CA1 region plays a crucial role in spatial memory formation, and its cellular composition, including astrocytes, is essential for synaptic plasticity and neuroprotection [7, 8]. Within this region, astrocytes are key players in synaptic plasticity and neuroprotection through their involvement in the tripartite synapse, where they modulate neurotransmission and structural plasticity [9, 10].

Environmental factors, including diet and chewing behavior, are linked to brain plasticity [11]. For example, masticatory stimulation has been shown to prevent memory impairment and preserve hippocampal function [1, 12]. Conversely, reduced masticatory activity from the consumption of soft diets or dental problems has been shown to accelerate cognitive decline in human beings, increasing the risk of dementia and impairing synaptic plasticity in animal models [13–16]. These findings underline the importance of chewing as a non-pharmacological intervention to mitigate age-related cognitive decline [17, 18].

Astrocytes undergo phenotypic changes during aging and are linked to age-related neurodegenerative diseases and associated cognitive impairments in later life [19–21]. These age-related changes include reduced branching and alter structural complexity, which impair the ability of an astrocyte to support neurons and maintain homeostasis [22–25]. Studies indicate that dietary factors, including masticatory activity, may influence astrocyte morphology within specific hippocampal layers, potentially impacting age-related impairments in spatial learning and memory [14, 26]. However, the specific impact of alternating dietary regimens on astrocyte morphology of CA1 and cognitive outcomes remains poorly understood [27].

The temporoammonic (TA) projection, a unidirectional excitatory pathway originating from the entorhinal cortex (layer III) and terminating primarily in the distal dendritic regions of CA1 pyramidal neurons within the hippocampus [28–30], plays a critical role in spatial and episodic memory by conveying processed sensory information from the cortex to the hippocampus. This pathway bypasses the traditional trisynaptic circuit and has distinct functional and anatomical properties that are vital for memory processing [28, 29, 31]. Loss of TA input impairs episodic memory and temporal discrimination, as CA1 neurons must then rely solely on CA3 inputs, which limits the ability of the hippocampus to update memory representations, resulting in rigid behavioral patterns and impaired learning [28, 29]. Notably, the disruption of this projection has been linked to Alzheimer’s disease and other neurodegenerative conditions, as CA1 is an area of early pathological involvement. Dysfunctional TA input could exacerbate deficits in early memory processes, such as mild cognitive impairment (MCI) [32, 33].

Astrocytes within the stratum *lacunosum-moleculare* (LM layer) are well positioned to regulate synaptic function by modulating neurotransmitter uptake and supporting synaptic homeostasis that are processes critical for memory consolidation [8, 34, 35]. Transitioning from a hard diet (HD) to a soft diet (SD) mimics real-world scenarios where abrupt lifestyle changes, such as dental issues or dietary modifications, disrupt normal oral activity [36]. Understanding these dynamics offers new insights into how environmental factors affect astrocytic function and suggests potential non-pharmacological interventions to maintain cognitive health.

This study considered the hypothesis that age-related morphological changes in astrocytes within the SLM are associated with cognitive decline and that maintaining masticatory activity may modulate these changes.

## Methods

### Ethical considerations and experimental groups formation

This study was approved by the Animal Research Ethics Committee (CEPAE) of the Biological Sciences Institute, Federal University of Pará (UFPA) (CEPAE-UFPA: 223-14) and conducted following the ARRIVE guidelines. The animals were provided with food and water ad libitum, maintained in a controlled environment at 23 ± 1 °C, and subjected to a 12-hour light/dark cycle.

Female adult female Swiss albino mice *(Mus musculus)* were obtained from pregnant females provided by the animal facility of the Instituto Evandro Chagas, with small differences in gestational age, allowing deliveries to occur on close dates. On postnatal day 5, six female pups from each litter were randomly selected and maintained with their dam until postnatal day 21 for breastfeeding.

The standardization of six pups per dam aimed to minimize potential adverse effects on growth and nervous system development by reducing stress related to competition for food and its possible behavioral and neurobiological consequences associated with larger litter sizes.

Each young group (6 M) initially consisted of 10 animals, whereas each aged group (18 M) consisted of 12 animals (Suppl. Table S1). Following animal losses and the selection of animals exhibiting consistent learning performance, five animals per group were included in the final analysis, as previously described by Siqueira-Mendes et al. [14]. The mice were selected on the sixth postnatal day, nursed until the 21st day, and then divided into six experimental groups based on age (6 and 18 months) and masticatory activity. Mice were assigned to three dietary regimes to analyze masticatory influence [36].

- *Hard Diet (HD)*: Pelleted food throughout.
- *Hard/Soft Diet (HD/SD)*: Pelleted food for half the time, followed by powdered food.
- *HD/SD/HD*: Alternating every two months between pelleted, powdered, and pelleted food to mimic rehabilitation.

In order to minimize potential systemic influences, such as metabolic or behavioral alterations that could interfere with the study outcomes, all experimental diets were standardized to ensure identical nutritional composition and caloric value, differing solely in consistency and form of presentation. Therefore, the only experimental variable manipulated in this study was the masticatory demand required to process the diet.

### Immunohistochemistry and 3D astrocyte reconstruction

At the end of the experimental period, all animals were weighed and subsequently anesthetized via intraperitoneal injection with a ketamine solution (90–120 mg/kg) combined with xylazine (5–10 mg/kg).

Thereafter, transcardial perfusion was performed. Following thoracotomy to expose the heart, a small incision was made in the right atrium, and the animals were perfused through the left ventricle, thereby reaching the systemic circulation, using a peristaltic pump. Initially, 0.9% sodium chloride solution containing sodium heparin (5000 IU/mL; 1 mL heparin per 1 L saline solution) was administered for approximately 10 min to remove circulating blood.

Subsequently, perfusion was continued with a 4% paraformaldehyde fixative solution prepared in 0.2 M phosphate buffer (pH 7.2–7.4) for approximately 30 min. Following fixation, craniotomy was performed for brain removal, and the tissue was sectioned into serial anatomical sections parallel to the transverse plane at a thickness of 60 μm using a vibrating microtome (Microm^®^ HM 650). All sections were collected and organized into wells at a 1:6 interval.

All sections obtained by vibratome microtomy were coded to ensure blinded analyses in subsequent procedures, thereby preventing identification of the experimental groups to which each animal belonged. One out of every six sections, maintaining a constant thickness of 60 μm, was selected for immunolabeling using a monoclonal antibody against glial fibrillary acidic protein (GFAP) (mouse anti-GFAP MAB360, Chemicon Int., USA).

Three-dimensional astrocyte reconstructions were performed using Neurolucida^®^ software (MBF Bioscience Inc., Frederick, MD, USA), installed on a computer connected to a NIKON Eclipse 80i microscope (Nikon, Japan) equipped with a motorized stage (MAC6000, Ludl Electronic Products, Hawthorne, NY, USA).

Initially, the stratum lacunosum-moleculare (SLM) of the hippocampal CA1 region was identified using the hippocampal sulcus as an anatomical landmark under a low-magnification objective (4×; Nikon, NA 0.13, DOF = 17.2 μm), ensuring accurate regional localization and minimizing observer ambiguity during cell selection. Furthermore, the SLM was distinguished from the *stratum radiatum* based on its stronger GFAP-immunolabeled background and the characteristic morphology of its astrocytes, which appeared smaller, exhibited shorter processes, and showed denser GFAP immunoreactivity. The identification of hippocampal subfields was facilitated by the cytoarchitectural differences between CA1 and CA3. Specifically, CA1 was recognized by its densely packed and well-organized pyramidal cell layer, whereas CA3 displayed a more dispersed and disorganized arrangement of pyramidal neurons, lacking the compact pyramidal cell packing readily observed in CA1 Astrocyte reconstructions were subsequently performed using a 100× objective (Nikon, NA 1.3; DOF = 0.16 μm).

After identifying the region of interest in each animal, namely the *stratum lacunosum-moleculare* of the CA1 region, its contour was delineated and the total area available for reconstruction was obtained for each animal. Subsequently, 30 cells per animal were digitally reconstructed. Prior to reconstruction, however, the number of cells to be reconstructed per section was calculated according to the proportional area of each section.

$$\mathrm{Q} \approx (\mathrm{Ax} \times 30)/\left(\mathrm{A1} + \mathrm{A2} + \mathrm{A3} + \ldots + \mathrm{Ax}\right)$$

Where:

Q = number of reconstructed cells; A = area of a given section; x = section for which the number of cells to be reconstructed was estimated.

After calculating the proportionality for each animal to determine the number of cells to be reconstructed per section, the cells selected for reconstruction were chosen using systematic random sampling. This sampling procedure was performed using a grid-and-box system. Briefly, a grid system (120 × 120 μm), represented in orange, was distributed throughout the region of interest. Within each grid, a green box (50 × 50 μm) was positioned in the upper-left region to delimit the astrocyte reconstruction area (Supplementary Fig. S1).

Each astrocyte was preferentially reconstructed within the area delimited by the green boxes, corresponding to the *SLM*. Cell selection followed predefined inclusion and exclusion criteria to ensure morphological integrity and analytical standardization. As an inclusion criterion, priority was given to astrocytes centrally located within the selected region of interest. Exclusion criteria included: [1] astrocytes displaying truncated processes resulting from immunohistochemical processing or anatomical sectioning; [2] cells exhibiting incomplete immunolabeling; and [3] astrocytes lacking vascular association. These criteria were established to minimize reconstruction artifacts and ensure the reliability and consistency of the three-dimensional morphological analyses. 30 cells per animal were reconstructed, totaling 877 reconstructed astrocytes. 23 cells were identified as outliers according to predefined statistical criteria and were excluded from th final analysis to prevent undue influence on the distribution of morphometric measurements.

### Behavioral assessment: morris water maze (MWM) test

We reassessed behavioral data from previous research by our group, conducted by Siqueira-Mendes et al. [14], focusing on the differences between the test days for each dietary regimen, correlating them with the morphometric analysis that is the focus of the present study [14]. The Morris water maze (MWM) test was used to assess spatial memory, and all experimental groups were trained according to the protocol originally proposed by Morris (1984) and adapted for mice.

A circular pool measuring 101 cm in diameter was used and filled with water maintained at a temperature of 22 ± 2 °C. The water level was adjusted to allow complete submersion of a circular escape platform measuring 22 cm in height and 13 cm in diameter, which was positioned 1 cm below the water surface. To conceal the platform, the water was made opaque using a non-toxic black dye.

The pool was virtually divided into four quadrants based on the cardinal points (North, South, East, and West). At the beginning of each trial, the animal was gently placed into the water facing the wall of the pool from one of the quadrants, which was randomly selected. The escape platform was located at the center of one predetermined quadrant and remained in the same position throughout the acquisition phase.

Three permanent external visual cues were positioned around the testing room and remained unchanged throughout all testing days, serving as spatial reference points to facilitate navigation toward the hidden platform.

These visual cues are relevant because spatial tasks may be performed using egocentric strategies (e.g., vestibular or proprioceptive signals) or allocentric strategies, which require the encoding of stable environmental features independent of the individual’s current position in space. In the latter case, the formation of a cognitive map of the environment is required.

After the adaptation phase, the animals were tested over the following five days, once a day in three consecutive trials. Each trial lasted a maximum of 60 s. For each of the three daily attempts, and on each test day, new entry points were randomly selected, while avoiding repeating the entry at the same point twice consecutively. In this phase, the platform location was randomly defined at the beginning of testing and remained unchanged throughout the five days. If the animal failed to locate the platform within the allotted time, it was gently guided to the platform. After reaching the platform, the animal remained there for 15 s.

Once the tests were completed and the recordings were obtained, by stopwatching. The ANY-maze Video Tracking System (StoeltingCo©) computer program was used to process and analyze behavioral parameters detected in the videos. Based on daily averages of escape latencies, a learning index (learning rate) was calculated to quantify relative improvement in performance across testing days. This index was calculated by the ratio between the escape latencies of the first day of testing (disregarding the adaptation phase) and the escape latencies of the subsequent days (second to fifth days) of testing, based on the following equation:

$$\mathrm{IC} = (\mathrm{L1}-\mathrm{LN})/(\mathrm{L1}+\mathrm{LN}),$$

L1 is the first-day latency, and LN is the latency on subsequent days. IC > 0 indicated improved performance, while IC ≤ 0 indicated no improvement. This contrast index is detailed described by Diniz et al. [37].

### Statistical analysis

The Shapiro–Wilk test was used to assess data normality. For the analysis of all morphometric variables (Suppl. Table S1), in both dorsal and ventral regions and according to each dietary condition, either the Student’s t-test or the Mann–Whitney test was applied, depending on data distribution.

Given the large number of variables evaluated in this study, it was necessary to identify, within each experimental group, those exhibiting a multimodal distribution (i.e., presenting at least two modes) (Schweitzer and Renehan, 1997). Briefly, among the 20 morphometric variables analyzed, variables displaying multimodal characteristics were identified through calculation of the Multimodality Index (MMI). The MMI was estimated based on the skewness and kurtosis of each sample (150 cells per experimental group) according to the following equation: MMI = (S² + 1)/{C + 3[(*n* − 1)²/((*n* − 2)(*n* − 3))]}, where MMI is the Multimodality Index, S is skewness, C is kurtosis, and n is the number of cells. Variables with MMI values greater than 0.55 were considered multimodal and were therefore selected for subsequent analyses.

After selecting the morphometric variables, hierarchical cluster analysis was performed using Ward’s linkage method and Euclidean distance on standardized variables. This approach was employed to classify astrocytes according to their morphology and to determine the number of distinct morphological phenotypes identified based on the selected morphometric parameters.

Subsequently, the same dataset was subjected to discriminant analysis, a multivariate statistical technique used for the classification and discrimination of objects. In the present study, discriminant analysis was used to identify which morphometric variables contributed most strongly to the formation of the clusters generated by the hierarchical analysis. All statistical analyses were performed using Statistica 7.1 (StatSoft Inc., Tulsa, OK, USA) and Jamovi (Version 1.6).

Regarding phenotype representation, after cluster analysis was performed to classify the phenotypes identified in each experimental group, validation of the selected Euclidean distance was conducted based on the following criteria: Mahalanobis distance index, canonical variable analysis, and Wilks’ lambda distribution analysis. This stage was performed using STATISTICA 12.0 software.

To validate the number of identified phenotypes (AST1, AST2, and AST3), the Shapiro–Wilk test was initially applied to verify normality, followed by either the Kruskal–Wallis test or one-way Analysis of Variance (ANOVA), as appropriate.

**Fig. 1:**
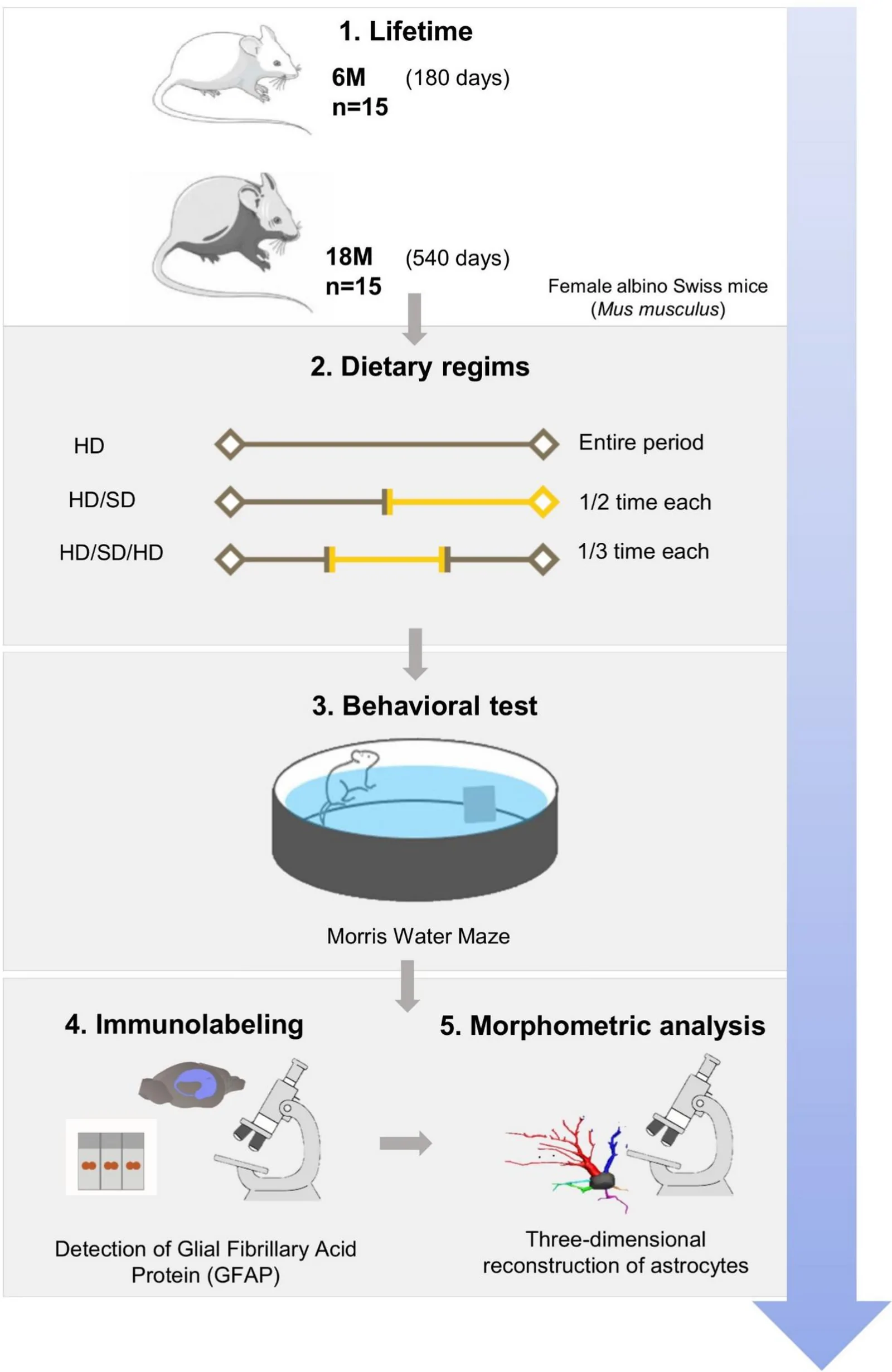
Overview of the Experimental Model in Mice. Mice were divided by age into two age groups—6 months (6 M) and 18 months (18 M) (*N* = 15 for each age)—and subjected to three dietary regimes: Hard Diet (HD), Hard Diet/Soft Diet (HD/SD), and Hard Diet/Soft Diet/Hard Diet (HD/SD/HD). Behavioral assessments were performed using reanalyzed data from Siqueira-Mendes et al. (2019). After the experimental period, the animals were perfused and euthanized, and their brains were collected for immunostaining with an anti-GFAP antibody. Three-dimensional reconstructions of astrocytes and morphometric analyses were performed to investigate the effects of diet and aging on astrocyte morphology and cognitive function (See Supplementary Table S1 for all morphometric features)

Furthermore, to evaluate the interaction between masticatory activity and age, two-way ANOVA followed by Tukey’s post hoc test was used. Data were processed using Statistica 7.1 and Jamovi software (v1.6). The sequence depicted in Fig. 1 ensures a logical progression through the experimental procedures, from dietary intervention and behavioral assessment to tissue collection, immunohistochemistry, and 3D astrocyte reconstruction. Each step builds on previous ones, contributing to a comprehensive understanding of how masticatory activity and aging influence spatial learning, memory, and astrocyte morphology. The diagram in Fig. 1 captures the experimental workflow, illustrating how diet, aging, and astrocyte morphology interconnect in the broader framework of the research approach. For behavioral analysis, a two-way ANOVA was performed.

## Results

### Morphometric differences in astrocytes across diets and age groups

**Fig. 2:**
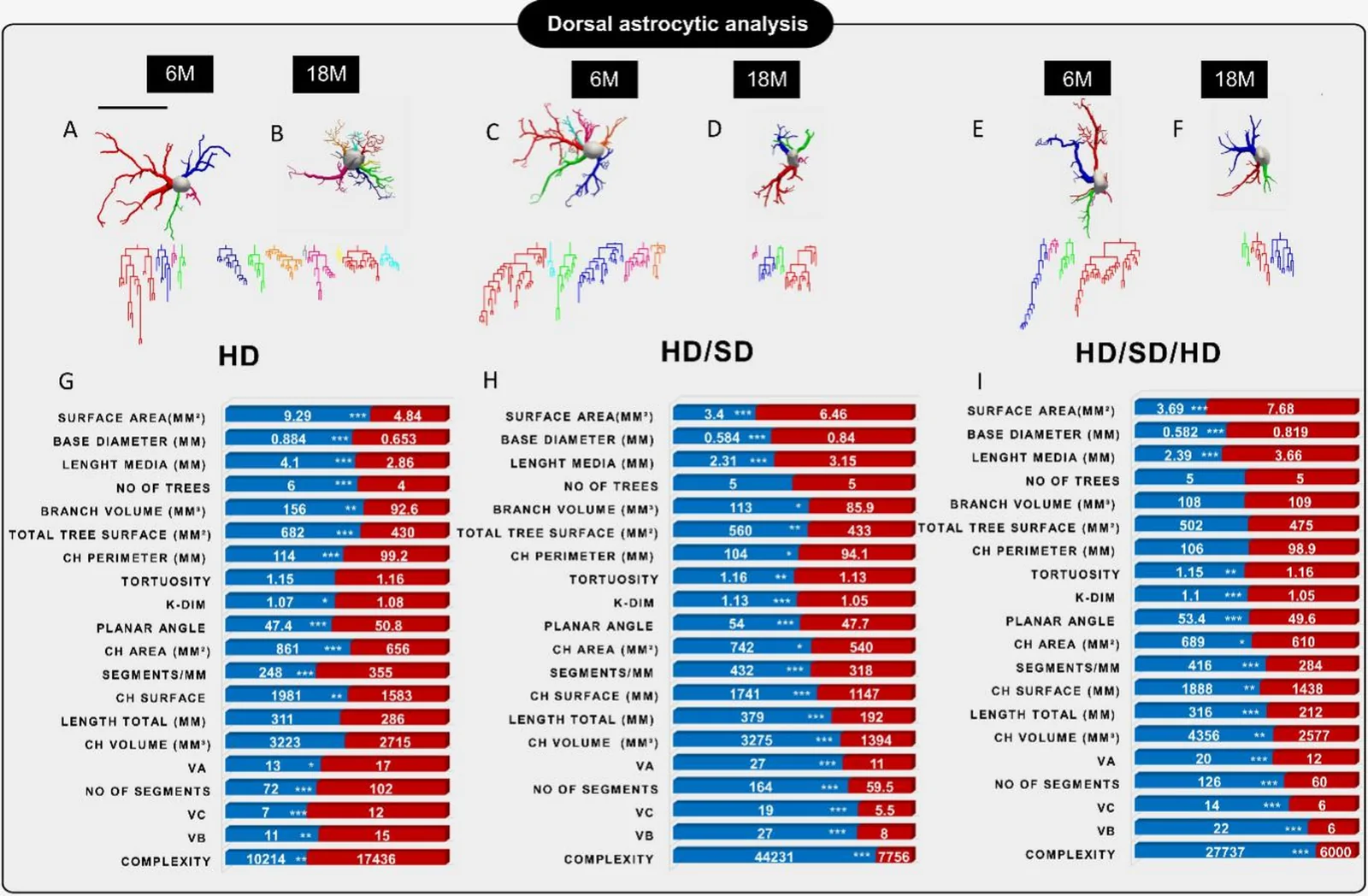
Aging Reduces Astrocyte Complexity in the Dorsal CA1 Region, Modulated by Chewing Regimen. Morphometric analysis reveals age- and diet-dependent reductions in astrocyte complexity in the dorsal CA1 region. Panels A–F present three-dimensional reconstructions of astrocytes from young (6 M) and aged (18 M) mice, comparing the Hard Diet (HD) (**A**, **B**), Hard Diet/Soft Diet (HD/SD) (**C**, **D**), and Hard Diet/Soft Diet/Hard Diet (HD/SD/HD) (**E**, **F**) regimens. Quantitative comparisons across 20 morphometric parameters for each diet are provided in panels G–I, where blue bars represent young animals and red bars represent aged animals. The results show a notable decline in complexity with age, with HD/SD/HD showing the highest complexity at 6 months. The data are color-coded to reflect the changes between 6 M (blue) and 18 M (red), with significant differences denoted by asterisks (e.g., ***p < 0.001, **p < 0.01). Scale bars: 25 μm. Abbreviations: HD (hard diet/pellet diet), HD/SD (pellet diet followed by soft diet/powdered diet), HD/SD/HD (pellet diet followed by powdered diet, followed by pellet diet again), VA (Vertex Analysis A), VB (Vertex Analysis B), VC (Vertex Analysis C), CH (Convex-Hull)

**Fig. 3:**
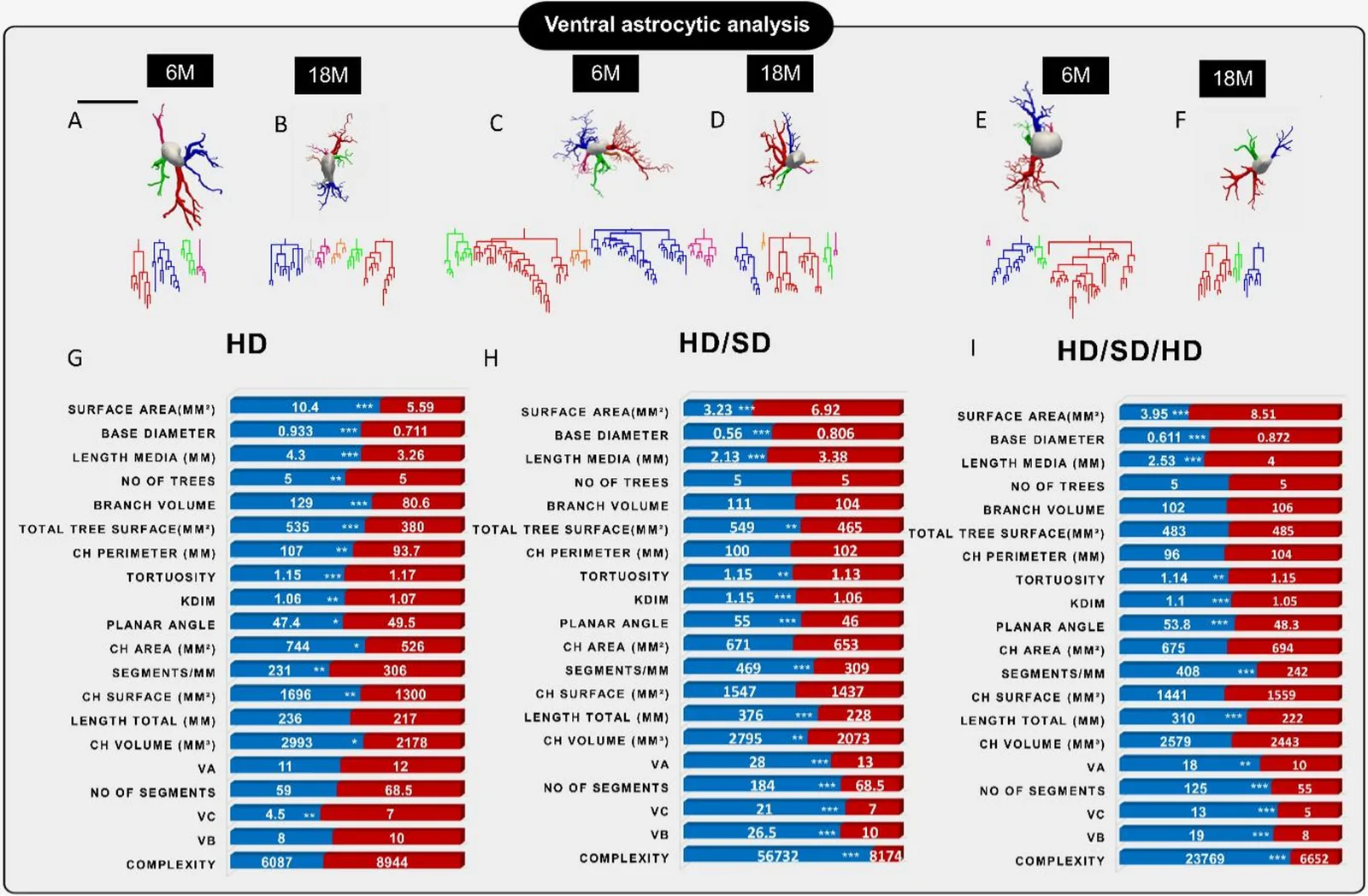
Astrocyte Complexity in the Ventral CA1 Region Declines with Age, with Variation Across Diets. Three-dimensional reconstructions and morphometric comparisons reveal diet- and age-dependent changes in astrocyte complexity in the ventral CA1 region. Panels A–F display astrocytes from young (6 M) and aged (18 M) mice for each chewing regimen: Hard Diet (HD) (**A**, **B**), Hard Diet/Soft Diet (HD/SD) (**C**, **D**), and Hard Diet/Soft Diet/Hard Diet (HD/SD/HD) (**E**, **F**). Quantitative morphometric data, shown in panels G–I, indicate reductions in astrocyte complexity with age, moderated by diet. The HD/SD regimen appears to partially preserve complexity in aged animals. The data are color-coded to reflect the changes between 6 M (blue) and 18 M (red), with significant differences denoted by asterisks (e.g., ***p < 0.001, **p < 0.01). Scale bars: 25 μm. Abbreviations: HD (hard diet/pellet diet), HD/SD (pellet diet followed by soft diet/powdered diet), HD/SD/HD (pellet diet followed by powdered diet, followed by pellet diet again), VA (Vertex Analysis A), VB (Vertex Analysis B), VC (Vertex Analysis C), CH (Convex-Hull)

3D morphological analysis of reconstructed astrocytes from the dorsal and ventral regions of *stratum lacunosum-moleculare* in the CA1 hippocampal region further underscores the effects of age and diet on astrocytic complexity at 6 months (6 M) and 18 months (18 M) of age for each chewing regimen (Figs. 2 and 3 for representative examples). Three-dimensional reconstructions of astrocytes in the dorsal hippocampus reveal a reduction in structural complexity with aging, which varies by diet. Young mice on the HD/SD/HD regimen exhibit the highest astrocytic complexity (*p* < 0.001) with a median score of 27,734, while aged animals show significant reductions, particularly in number of segments, length total, Convex-Hull Volume, among others. (Fig. 2, panels G-I) (See Supplementary Table S2–S4 to Dorsal CA1 Region).

The HD/SD regimen appears to moderate these age-related declines, maintaining a higher degree of astrocytic complexity compared to the other regimens (*p* < 0.001).

In the ventral hippocampus, astrocytic complexity similarly declines with age, but the HD regimen partially preserves complexity in aged animals (6 M: 6087; 18 M: 8944 medians scores, *p* = 0.106) contrasting with more substantial reductions in the HD/SD and HD/SD/HD groups. The 3D reconstructions of astrocytes at 6 months (6 M) and 18 months (18 M) of age for the following chewing regimens highlight astrocytic structural changes, including branching and overall complexity variations, and how they are influenced by age and chewing pattern (Fig. 3, panels A-F). These differences across diets highlight the potential influence of chewing patterns on maintaining astrocytic morphology with age (Fig. 3, panels A-I). A quantitative comparison of astrocytic morphology for each regimen: HD (G), HD/SD (H), and HD/SD/HD (I) more clearly defines the differences (Fig. 3, panels G-I) using 20 morphometric variables. The data reveal that astrocytic complexity tends to decrease with aging, particularly in the HD/SD and HD/SD/HD regimens, as shown by reductions in length total, number of segments, and vertex analysis at 18 months in these diets. Interestingly, the HD regimen (G) shows some preservation of complexity with age compared to the other regimens. These findings reveal that the chewing regimen and age influence astrocyte structural integrity and complexity, potentially impacting neural function in the ventral brain region (See Supplementary Table S6–S8 to Ventral CA1 Region).

### Cluster analysis and phenotypic classification

**Fig. 4:**
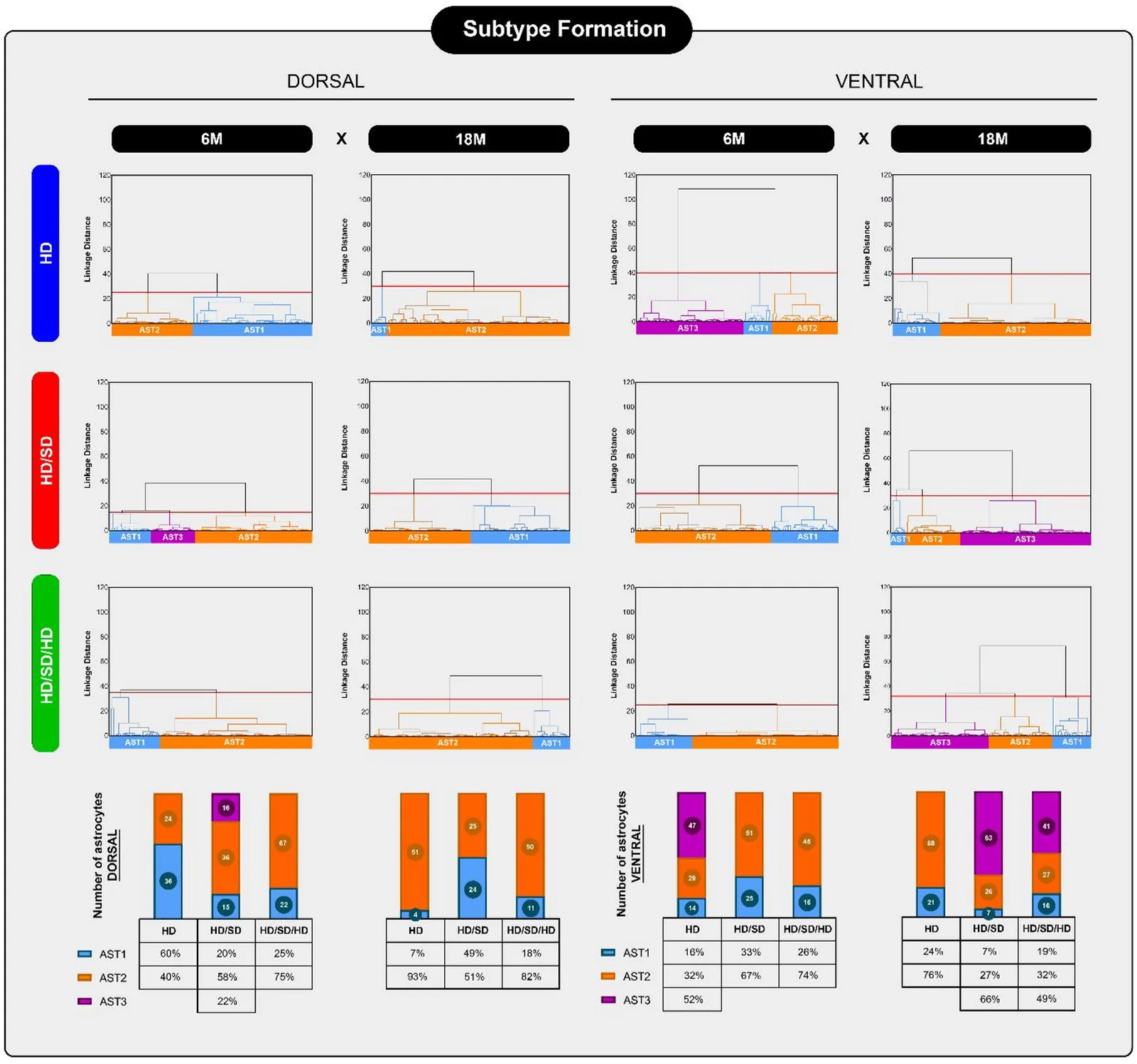
Chewing Regimen and Age Affect Astrocyte Complexity and Subtype Distribution. Cluster analysis identifies distinct astrocyte subtypes (AST1, AST2, and AST3) across different diets and brain regions in aged animals, which presents the astrocyte complexity of each subtype in dorsal and ventral regions, with AST1 showing the highest complexity in younger animals. Below the clusters, the figure displays the distribution of astrocyte subtypes across diets, showing a shift from high-complexity (AST1) to low-complexity (AST3) astrocytes with aging. Statistical comparisons highlight significant complexity differences between astrocyte subtypes and the differential impact of age and diet on astrocyte subtype distribution

Morphological complexity was the morphometric variable that contributed most strongly to the identification of astrocytic morphotypes, as revealed by the cluster and discriminant analyses. Figure 4 provides stacked bar graphs showing the distribution of astrocyte subtypes (AST1, AST2, and AST3) in young and aged animals. Each graph shows the proportion and total number of astrocytes for the dorsal and ventral regions under each chewing regimen. In the dorsal area, AST1 dominates in younger animals, particularly in the HD/SD/HD regimen. However, with aging, there is a shift toward AST2, especially in HD and HD/SD. Compared to the dorsal region, a more pronounced shift toward AST3 is observed in the ventral region with aging, particularly under the HD/SD and HD/SD/HD regimens. This suggests a differential impact of aging on astrocyte subtype distribution between brain regions.

**Fig. 5:**
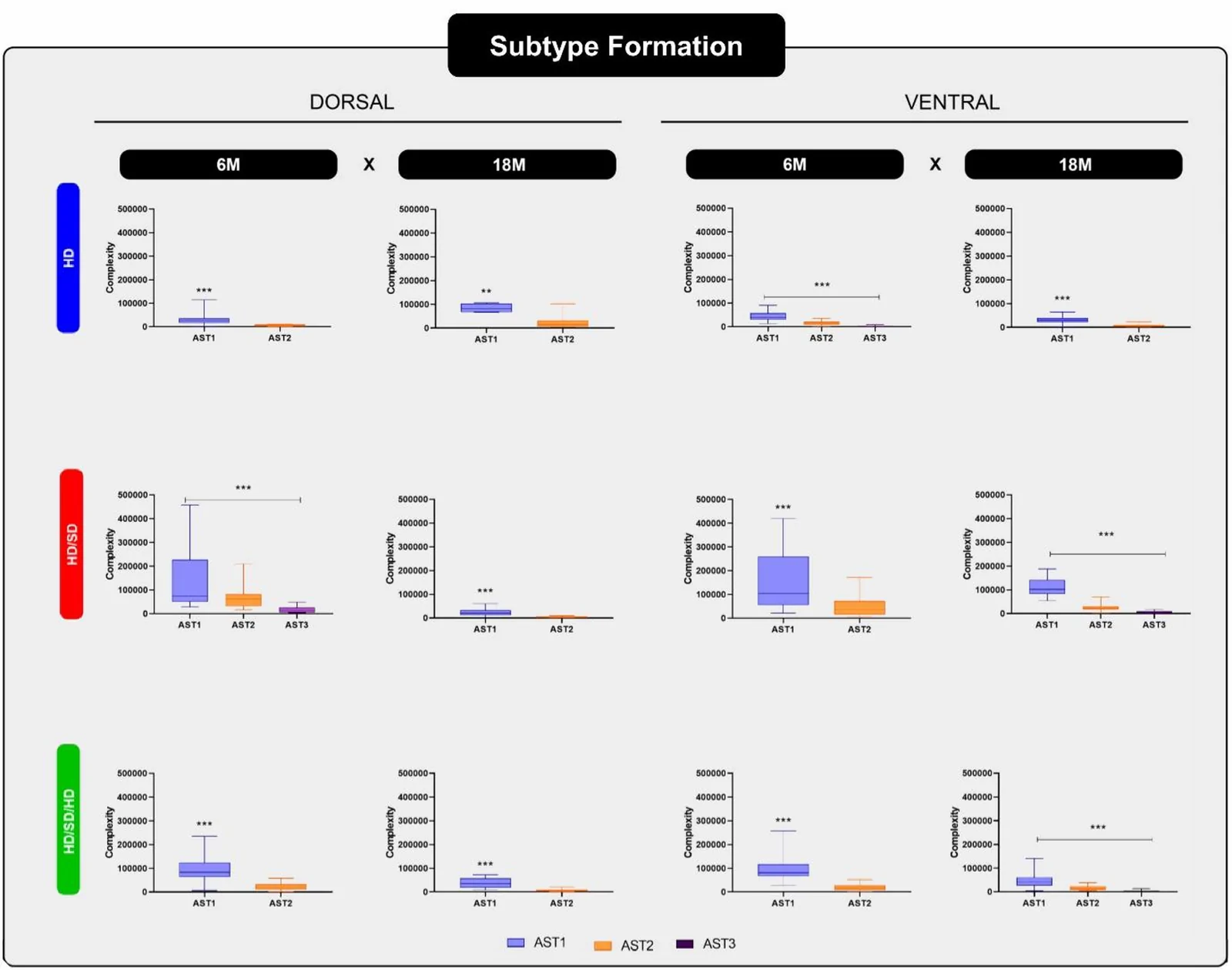
Statistical comparisons highlight significant complexity differences between astrocyte subtypes. Formation of AST1, AST2 and AST3 cellular phenotypes found in the SLM in CA1 of young (6 M) and senile (18 M) animals in different regimes of masticatory activity. The values were compared using the Mann-Whitney and Kruskal-Wallis tests

Astrocytes were classified into subtypes based on complexity (AST1, AST2, and AST3), and analysis reveals distinct age- and region-dependent distributions. AST1, the most complex subtype, is prevalent in younger animals and primarily in the dorsal region, while AST3, a low-complexity subtype, dominates in aged animals and is more prominent in the ventral region. This shift from high- to low-complexity astrocytes with aging suggests that astrocyte complexity may be a key factor in cognitive resilience, particularly in relation to diet (Fig. 4). Notably, animals on the HD/SD/HD regimen retain a higher proportion of AST1 astrocytes with age, suggesting a protective effect of this dietary pattern on astrocytic integrity. The box plots in Fig. 5 compare the complexity of astrocyte subtypes (AST1, AST2, and AST3) in the dorsal and ventral regions. Each row corresponds to a specific chewing regimen: HD (top), HD/SD (middle), and HD/SD/HD (bottom). As animals age, astrocytic complexity decreases across all subtypes, with AST2 and AST3 showing the lowest values, particularly in the ventral region.

### Detailed morphological analysis of low-complexity astrocytes (AST2)

**Fig. 6:**
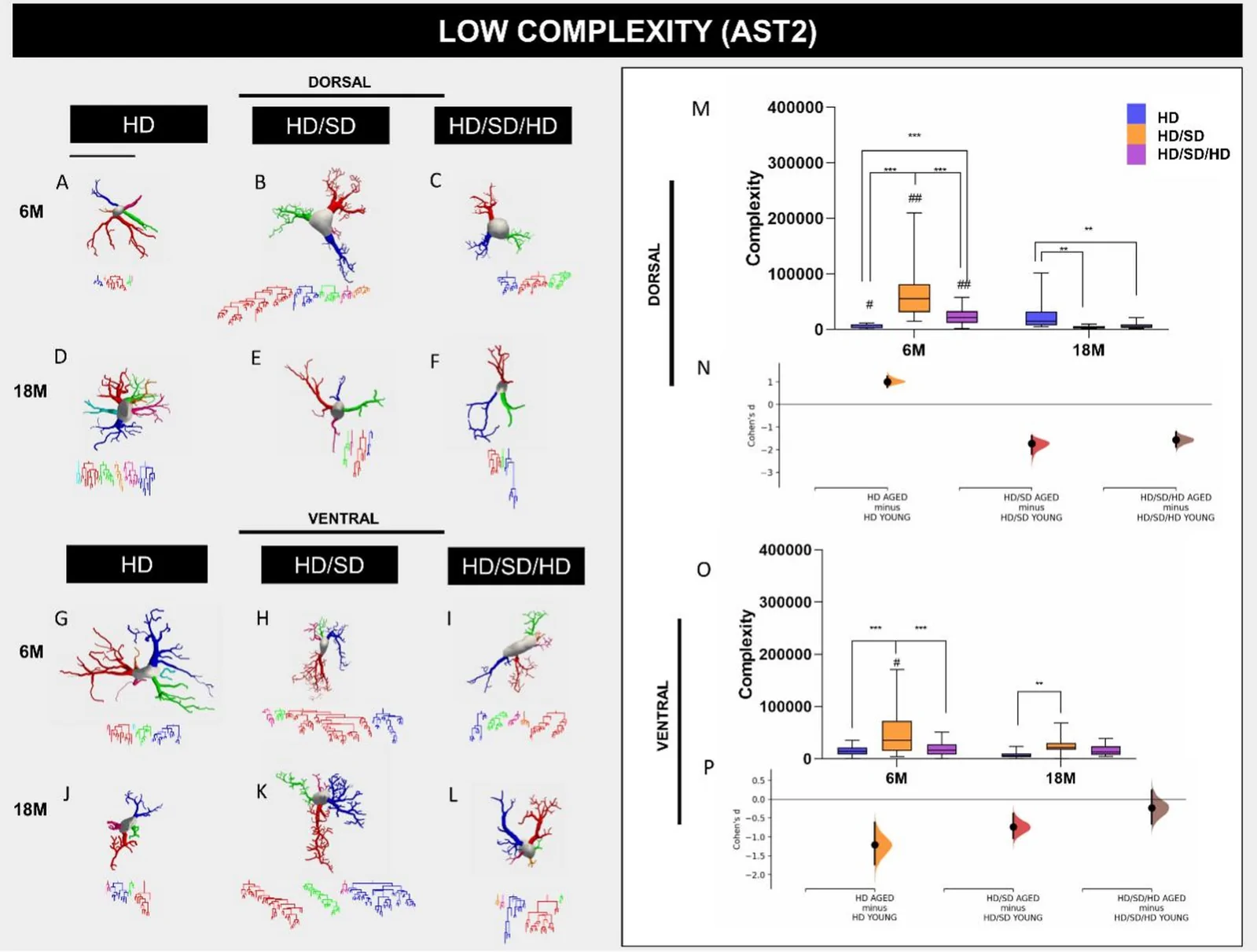
Aging and Diet Shape Low-Complexity Astrocyte (AST2) Morphology in the Dorsal and Ventral CA1 Regions. Morphological complexity of low-complexity astrocytes (AST2) across diets and ages in the dorsal (M) and ventral (O) regions shows diet- and age-dependent changes. Panels A-L provide three-dimensional reconstructions of representative AST2 astrocytes across dietary regimens (HD, HD/SD, HD/SD/HD) in both age groups. Quantitative comparisons confirm significant reductions in complexity with age, particularly under HD conditions, with the HD/SD diet mitigating some decline. Violin plots (N, P) illustrate effect sizes, highlighting the diet’s role in modulating astrocytic changes with aging

Further analysis focuses on the morphometric characteristics of low-complexity astrocytes (AST2) in both dorsal and ventral regions across age and dietary conditions. Aging consistently reduces branching and complexity in AST2 astrocytes, with marked differences between younger and older mice (Supplementary Table S9). Diet influences this decline, as younger mice on the HD/SD regimen exhibit the highest complexity, while aged mice show significant reductions across all diets, particularly in the dorsal region (Fig. 6, panels A-L). Statistical analysis of Cohen’s d effect sizes confirms that age-related changes in astrocytic complexity are most pronounced under the HD diet, though the HD/SD regimen mitigates this decline to some extent. Panels A through L provide three-dimensional reconstructions of astrocytes from mice subjected to one of three dietary regimens: hard diet (HD), HD followed by soft diet (HD/SD), and HD, followed by SD and followed by HD (HD/SD/HD). Each row compares these astrocytes across the two age groups (6 M and 18 M) in both the dorsal (A-F) and ventral (G-L) regions. These reconstructions highlight the reduction in astrocytic branching and complexity with age, highlighting pronounced differences between younger and older mice across diet conditions. Panels M and O quantify astrocytic complexity in the dorsal and ventral regions, respectively, showing significant differences between the age groups (need to make it clear too what *P*-value *** related). The younger mice consistently exhibit higher astrocytic complexity across all diet conditions, particularly in the dorsal region, with notable reductions in the 18-month group. The ventral region shows fewer age-related differences, but the overall trend of reduced complexity with aging is evident.

Panels N and P display violin plots of Cohen’s d effect sizes, comparing the impact of age on astrocyte complexity across dietary conditions. These plots confirm that the magnitude of age-related decline is diet-dependent, with the HD/SD regimen increasing complexity to some extent compared to the HD diet alone. This figure underscores the interaction between age, diet, and region-specific brain changes, providing insights into how dietary interventions may influence the structural integrity of astrocytes throughout aging.

### Comparative analysis of high-complexity astrocytes (AST1)

**Fig. 7:**
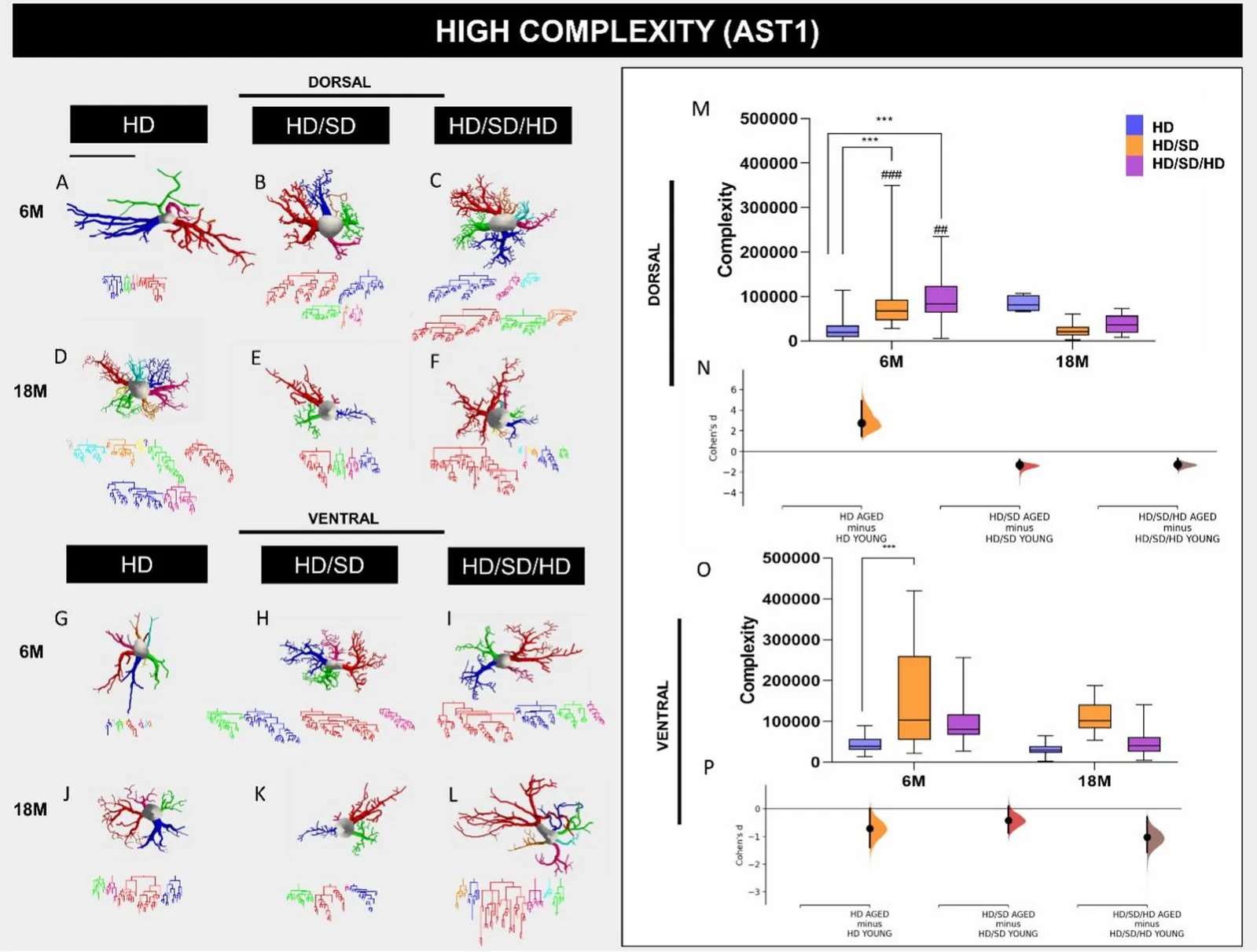
High-Complexity Astrocyte (AST1) Morphology is Maintained in Young Mice and Declines with Age Across Diets. Comparative analysis of high-complexity astrocytes (AST1) reveals distinct age- and diet-related effects on astrocyte morphology in the dorsal and ventral regions. Mean values and standard deviations of AST1 complexity across dietary regimens (HD, HD/SD, HD/SD/HD) are presented for both age groups. Panels M and O show two-way ANOVA comparisons, with age and diet significantly affecting astrocyte morphology. Three-dimensional reconstructions of representative astrocytes (**A**–**F** for dorsal, **G**–**L** for ventral) illustrate phenotypic clustering, emphasizing that diet modulates the age-dependent decline in complexity

Comparing AST1 astrocytes across young and aged animals shows significant effects of diet and age on astrocytic morphology. The HD/SD regimen shows the greatest preservation of astrocytic complexity with aging, highlighting the influence of dietary modulation on the resilience of high-complexity astrocytes (Fig. 7). These findings suggest that both age and diet significantly shape astrocyte morphology in the *lacunosum-moleculare* layer of CA1, with potential implications for cognitive health.

A two-way ANOVA was conducted to analyze the effects of diet, age, and their interaction on the complexity of high-complexity astrocytes (AST1) in the dorsal region of young and aged mice (Supplementary Table S10). The results show a significant interaction between age and diet (F = 12.414, *p* < 0.001), suggesting that the dietary effects on astrocyte morphology are age dependent. However, the main effect of diet was not significant (F = 0.618, *p* = 0.541 indicating that dietary changes alone do not significantly impact astrocytic complexity.

Young mice under a hard-diet (HD) regimen exhibited an astrocyte complexity of 24,364 ± 21,865, whereas aged mice under the same diet had a much higher complexity (83,417 ± 19,092). Although the difference between these groups was not statistically significant (*p* = 0.116), the effect size (Cohen’s d = 2.73) indicates a significant impact of aging under HD conditions. Dietary interventions: The comparison between the HD/SD (hard-diet followed by soft-diet) group in young and aged mice revealed a negative effect size (Cohen’s d = − 1.31), indicating a reduction in complexity with aging. Likewise, further dietary shifts in the HD/SD/HD group showed consistent but not significant differences across age groups (*p* = 0.473), with negative effect sizes (e.g., Cohen’s d = − 1.27). Within-group comparisons: While comparisons within the same age group (e.g., HD/SD 6 M vs. HD/SD/HD 6 M) did not reach significance (*p* = 1.0), there were significant differences between age groups within other dietary conditions. For example, young mice under the HD/SD diet displayed higher astrocytic complexity (96,652 ± 91,074) than their aged counterparts (24,302 ± 17,030, *p* < 0.001). Similarly, significant differences were found in other age-diet comparisons (e.g., HD/SD 6 M vs. HD/SD 18 M, *p* < 0.001).

These findings emphasize the complex interplay between aging and diet in shaping astrocytic morphology. While diet alone exerts minimal influence, its interaction with age plays a crucial role in modulating astrocytic complexity. The effect sizes further highlight the significance of age-related changes, especially under hard-diet conditions, suggesting that astrocyte complexity is sensitive to the natural aging process and an individual’s dietary history.

A comparative analysis of high-complexity astrocytes (AST1) across young (6 months) and aged (18 months) mice was performed using a two-way ANOVA, revealing significant effects of diet, age, and their interaction on astrocytic complexity (Fig. 7) (Supplementary Table S10). The interaction between age and diet was highly significant, with an F-value of 53.8 and a *p*-value < 0.001, indicating that the influence of diet on astrocyte morphology changes notably with aging. Cohen’s d values highlight substantial effect sizes, particularly when comparing different age groups and dietary conditions. Dietary impact: Diet alone produced a significant main effect (F = 19.9, *p* < 0.001), with high-complexity astrocytes showing distinct morphological patterns under different dietary regimens. Age comparisons: Notably, young mice (6 M) under hard-diet (HD) conditions exhibited a mean astrocytic complexity of 4980 ± 3637, whereas aged mice (18 M) under the same conditions displayed a much higher complexity of 22,735 ± 21,324 (*p* = 0.011). Combined diets and aging: The hard-diet-switch-to-soft-diet (HD/SD) group exhibited a mean complexity of 65,078 ± 45,249 in young mice and 4093 ± 2666 in aged mice, with significant differences across all comparisons (*p* < 0.001). Significant differences were observed between groups undergoing additional dietary modifications (HD/SD/HD) between age groups, suggesting that dietary manipulations modulate astrocytic complexity over time.

These findings suggest that dietary interventions and aging influence the morphological complexity of high-complexity astrocytes independently and through interaction. The effect sizes from specific group comparisons reinforce the strength of these changes, highlighting the significant roles of age and diet in shaping astrocyte morphology in the lacunosum molecular layer of CA1.

### Representative photomicrographs of GFAP-immunolabeled astrocytes in the temporoammonic pathway across different masticatory regimens

**Fig. 8:**
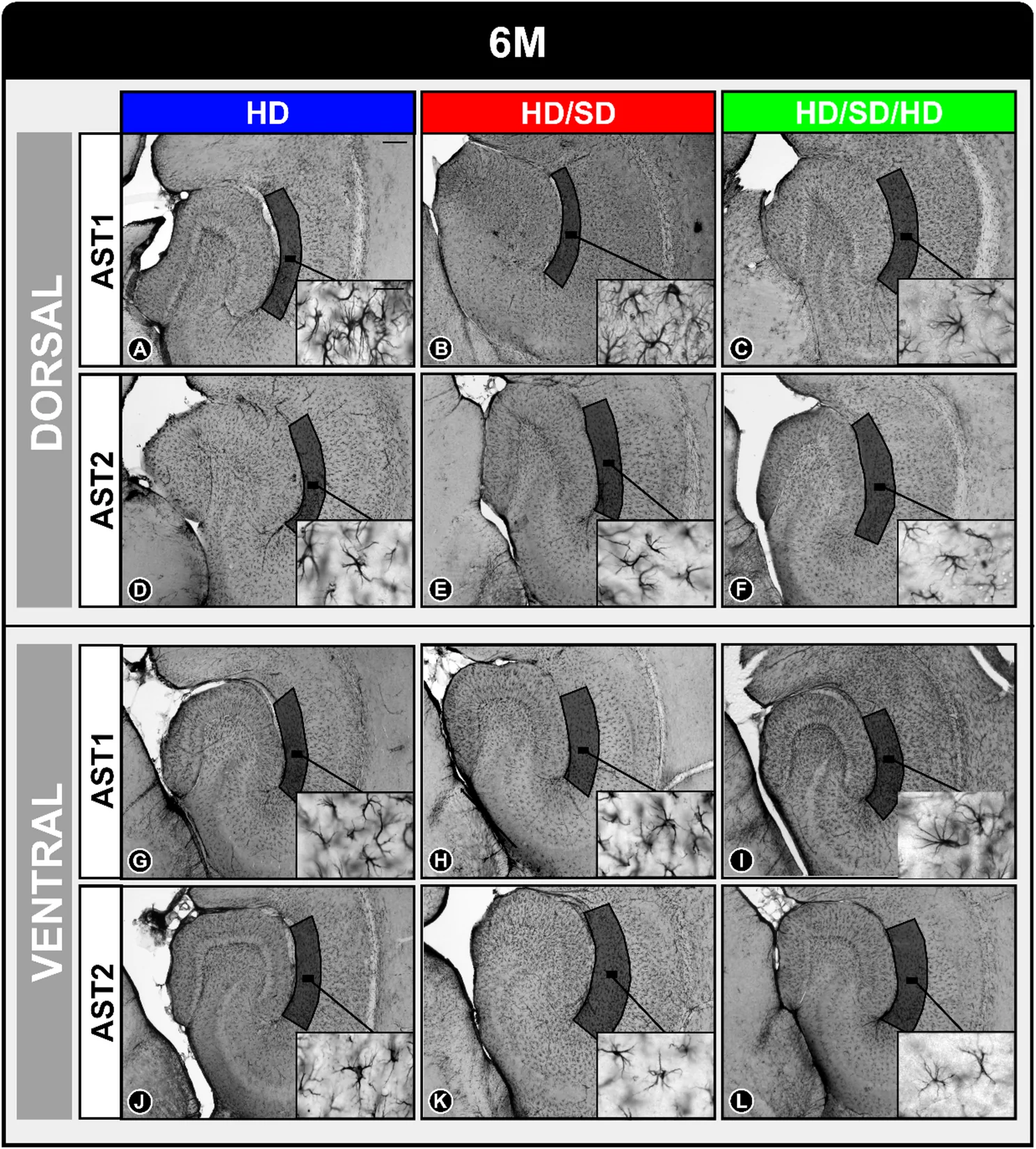
Representative photomicrographs of GFAP-immunolabeled astrocytes in the temporoammonic pathway of 6-month-old animals subjected to different masticatory regimens (HD, HD/SD, and HD/SD/HD). Panels A–F show the dorsal hippocampus, whereas panels G–L show the ventral hippocampus. The low-magnification photomicrographs (5×) identify the stratum lacunosum-moleculare, indicated by the shaded area, from which astrocytes were selected for analysis. Insets correspond to high-magnification photomicrographs (100×) illustrating representative AST1 and AST2 GFAP-immunolabeled astrocytes within the selected region. *The black square indicates the region shown at higher magnification in the inset*

**Fig. 9:**
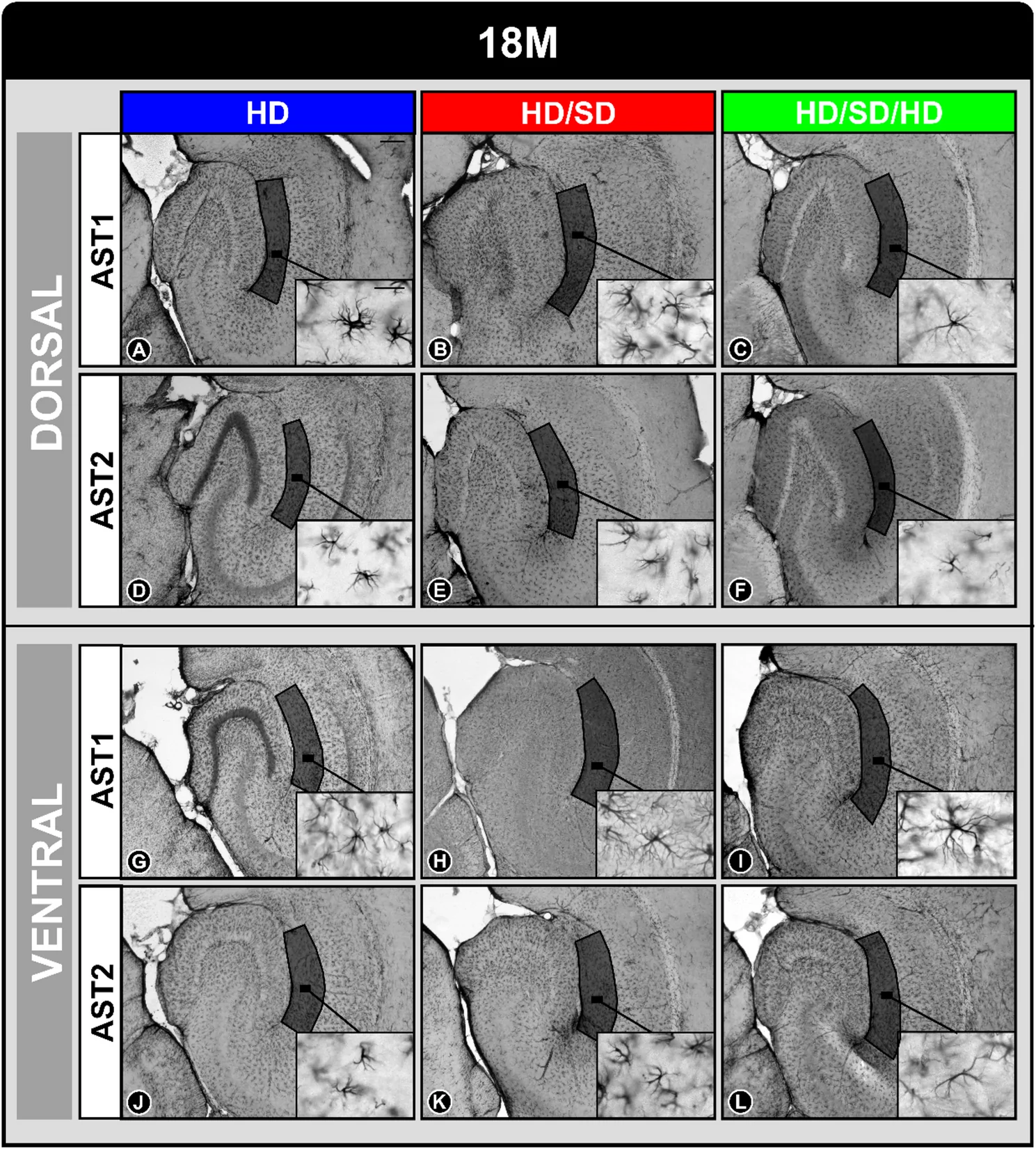
Representative photomicrographs of GFAP-immunolabeled astrocytes in the temporoammonic pathway of 18-month-old animals subjected to different masticatory regimens (HD, HD/SD, and HD/SD/HD). Panels A–F show the dorsal hippocampus, whereas panels G–L show the ventral hippocampus. The low-magnification photomicrographs (5×) identify the stratum lacunosum-moleculare, indicated by the shaded area, from which astrocytes were selected for analysis. Insets correspond to high-magnification photomicrographs (100×) illustrating representative AST1 and AST2 GFAP-immunolabeled astrocytes within the selected region. *The black square indicates the region shown at higher magnification in the inset*

In order to complement the quantitative morphometric analyses, representative photomicrographs of GFAP-immunolabeled astrocytes in the temporoammonic pathway at 6 months are presented in Fig. 8 and at 18 months in Fig. 9. Representative AST1 and AST2 astrocytes, from the dorsal and ventral hippocampus, are shown for each experimental group (HD, HD/SD, and HD/SD/HD). Higher-magnification insets highlight the morphology of the selected astrocytes, allowing qualitative visualization of the immunolabeled cells analyzed throughout the study.

### Dietary and age effects on spatial learning and memory

Based on the morphometric findings observed in the dorsal hippocampal region, we revisited the behavioral data reported by Siqueira-Mendes et al. [14] to investigate potential functional correlates of the structural alterations identified in the present study.

**Fig. 10:**
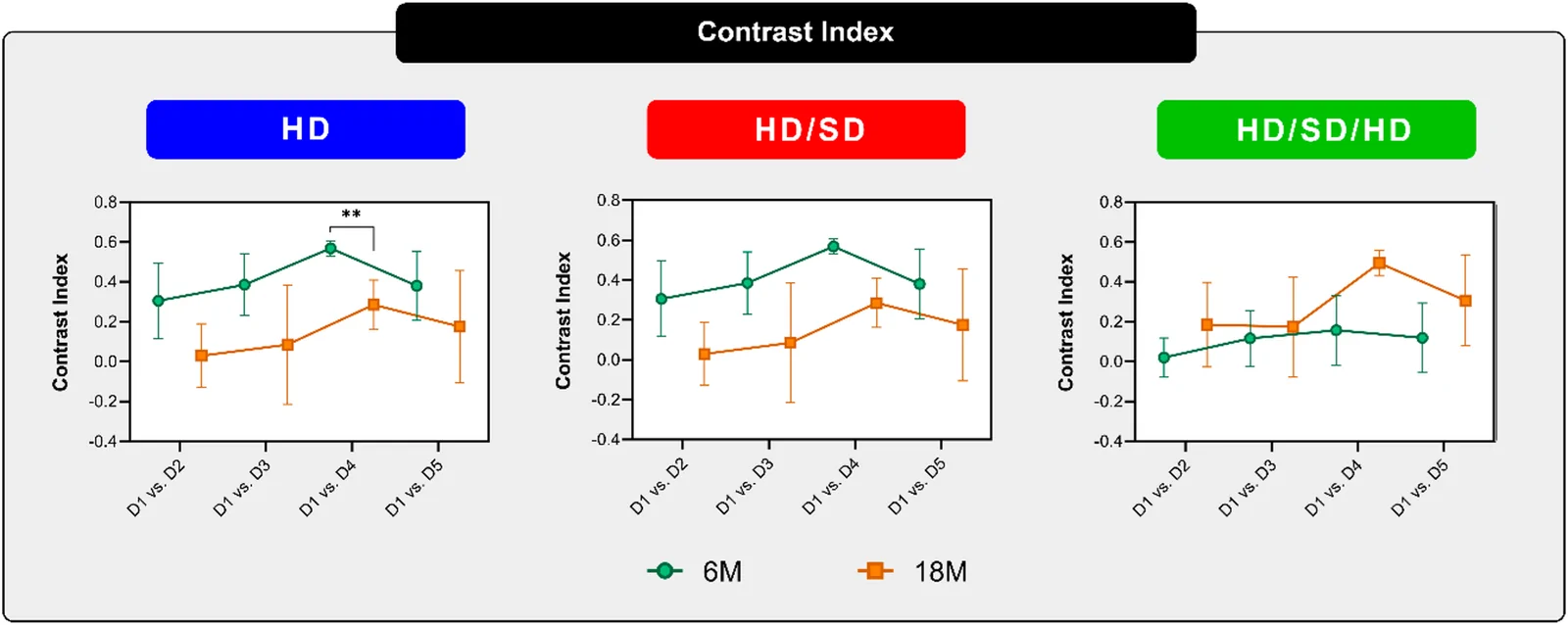
Age and Chewing Regimens Influence Spatial Learning and Astrocyte Complexity. Contrast index across training days in the Morris water maze for animals subjected to different masticatory conditions (HD, HD/SD, and HD/SD/HD). Data are presented as mean ± SD for 6-month-old (6 M) and 18-month-old (18 M) animals. For each dietary condition, statistical analysis was performed using repeated-measures two-way ANOVA, considering age as the between-subject factor and training day as the repeated-measures factor, followed by appropriate post hoc comparisons when applicable. *p < 0.05, **p < 0.01. Contrast index equation [C = (L1 - LN)/(L1 + LN)]

Our findings reveal a relationship between chewing regimen, age, and spatial learning performance, as measured by the Morris water maze over five days (Fig. 10). Learning rates, assessed across three dietary regimens—hard diet (HD), HD followed by a soft diet (HD/SD), and HD/SD/HD—demonstrate that cognitive improvement varies significantly with diet, as shown by learning performance differences across these regimens HD (Supplementary Information 1), HD/SD (Supplementary Information 2), HD/SD/HD (Supplementary Information 3).

In the HD group, young animals (6 M) exhibited consistently higher contrast index values across training days compared with aged animals (18 M), with a significant difference detected on D4 (**p < 0.01). While 6 M animals showed a marked increase in contrast index reaching a peak at D4, 18 M animals displayed a more modest progression across days. These findings indicate an age-related attenuation in learning progression under normal masticatory conditions. Notably, no significant age-related differences were observed in either the HD/SD or the rehabilitated HD/SD/HD groups.

## Discussion

The LM of the hippocampal CA1 region, a critical target of the TA pathway, is a valuable anatomical substrate for studying age-related morphological changes in astrocytes and their impact on cognitive decline. Astrocytes in this layer are strategically positioned to influence memory consolidation and spatial learning through their roles in synaptic plasticity, neurotransmitter uptake, and the maintenance of homeostasis—all essential processes for these cognitive functions. As astrocytic morphology is intricately linked to their functional capacity, exploring these cellular structures is crucial for predicting and understanding functional outcomes associated with aging and neurodegenerative diseases [38].

Here, we tested the hypothesis that age-related morphological changes in the *stratum lacunosum-moleculare* astrocytes affect cognitive performance. Our findings reveal a complex interplay between aging and diet in shaping astrocytic morphology and age-related cognitive decline. While aging was associated with reduced astrocyte complexity, in young mice especially, a soft diet increased astrocyte complexity, but this was accompanied by reduced cognitive performance. This somewhat counterintuitive result will be discussed below.

### Age-related astrocytic changes in the LM layer

Taken together, our findings suggest that the effects of masticatory activity on hippocampal morphology and memory processes may differ across stages of life. The more pronounced astrocytic and behavioral changes observed in young animals may reflect a greater responsiveness of neural circuits to environmental modulation, whereas aged animals may exhibit reduced adaptive plasticity. Thus, the impact of masticatory alterations appears to be age-dependent rather than uniform throughout the lifespan.

Aging induces significant structural modifications in astrocytes, including reductions in branching complexity, impaired calcium signaling, and diminished glutamate clearance, which collectively disrupt fundamental mechanisms such as long-term potentiation (LTP), essential for memory and learning [24, 25]. These changes are believed alter the ability of an astrocyte to efficiently support synaptic networks, compromising their metabolic function and contributing to neuroinflammation—a hallmark of cognitive aging. As astrocytes transition to a more reactive state with aging, they release pro-inflammatory cytokines, such as interleukin-1β (IL-1β) and tumor necrosis factor-alpha (TNF-α), further exacerbating neuronal dysfunction and fostering cognitive decline [10, 39].

In this sense, astrocytes play a central role in the regulation of synaptic function within the hippocampus through many mechanisms as neurotransmitter clearance, ion homeostasis, metabolic support, and modulation of synaptic plasticity. Therefore, morphological alterations in these cells may have important functional consequences. Reduced branching complexity and retraction of perisynaptic astrocytic processes can impair glutamate uptake and calcium signaling, leading to disruption of synaptic homeostasis and altered neuron–glia communication. Such dysfunction may compromise mechanisms underlying long-term potentiation (LTP), particularly in hippocampal regions involved in memory encoding and consolidation. In addition, impaired astrocytic support may affect the stability and efficacy of synaptic connections, contributing to reduced plastic adaptability of hippocampal circuits. Collectively, these alterations may ultimately manifest at the behavioral level as deficits in learning and memory performance, including impairments in spatial navigation, memory consolidation, and cognitive flexibility. Thus, astrocytic morphological remodeling may represent a mechanistic link between cellular alterations and hippocampus-dependent behavioral dysfunction [9, 10].

In addition, several studies have highlighted that with aging, astrocytes within the hippocampus display increased glial fibrillary acidic protein (GFAP) expression and they have been observed to retract their processes, which impairs their capacity to modulate synaptic activity and support plasticity, these changes are associated with decreased synaptic coverage, reduced glutamate clearance, and impaired K+ buffering [24, 25, 40]. Consequently, deficits in astrocytic support at the LM layer contribute to the breakdown of spatial and episodic memory systems, accelerating cognitive decline.

Moreover, environmental stressors, such as reduced masticatory activity or dietary changes, aggravate astrocytic dysfunction and cognitive impairments. Studies indicate that astrocytes within the LM layer are particularly vulnerable to these stressors, which exacerbate calcium imbalances, impair metabolic coupling, and intensify local inflammation [14]. Understanding these interconnected processes offers insights into the pathophysiology of age-related cognitive disorders. It highlights the potential for therapeutic strategies to preserve astrocytic function within the LM layer to support cognitive resilience.

### Astroglial heterogeneity and aging-related remodeling

Our findings suggest that astroglial aging in the *stratum lacunosum-moleculare* does not follow a uniform morphological trajectory, but instead occurs in a phenotype-specific manner modulated by masticatory activity.

In the AST1 phenotype, characterized by higher morphological complexity, aging alone did not significantly alter astrocytic complexity in animals maintained under normal masticatory conditions (HD group) in the dorsal region, since no differences were observed between young and aged animals. In contrast, animals exposed to masticatory dysfunction (HD/SD and HD/SD/HD groups) exhibited reduced complexity during aging, with young animals showing higher complexity than aged animals. Interestingly, among aged animals, no differences were observed between dietary conditions, suggesting that AST1 may represent a more stable and aging-driven astrocytic phenotype, with limited sensitivity to masticatory modulation during advanced aging.

Conversely, the AST2 phenotype, characterized by lower complexity, displayed a distinct response pattern. In animals maintained under physiological mastication (HD), aged animals exhibited greater complexity compared to young animals, suggesting the occurrence of adaptive or compensatory astroglial remodeling during physiological aging. However, this age-related increase was not observed in the masticatory dysfunction groups (HD/SD and HD/SD/HD), in which aged animals exhibited lower complexity compared to young animals. Furthermore, among aged animals, the HD group displayed greater complexity than both HD/SD and HD/SD/HD groups.

Together, these findings suggest that preserved masticatory activity may support adaptive astroglial remodeling during aging, whereas masticatory dysfunction may impair astrocytic plasticity and promote a maladaptive aging trajectory. Importantly, our results also reinforce the concept of astroglial heterogeneity, indicating that distinct astrocytic phenotypes may exhibit differential vulnerability to aging and environmental modulation [41].

Rather than representing contradictory findings, the coexistence of preserved, hypertrophic, or simplified astroglial morphologies may reflect distinct biological states associated with physiological aging, compensatory remodeling, or maladaptive responses induced by chronic masticatory dysfunction.

### Masticatory activity as a neuroprotective strategy

Dietary and environmental factors, such as masticatory activity, are increasingly recognized as non-pharmacological strategies to counteract age-related cognitive decline. Mastication provides sensory input that stimulates the hippocampus and other brain areas involved in memory and learning, enhancing synaptic plasticity and neurogenesis [1]. Studies have shown that the act of chewing not only improves hippocampal function but also reduces stress-related behaviors and supports neuroplasticity, which is crucial for memory consolidation [2, 17]. Alternating dietary regimens that mimic real-life fluctuations—such as the transition from hard to soft diets and back—reveal the plasticity of astrocytes in response to environmental changes. This study is in keeping with findings that masticatory behavior preserves hippocampal integrity, reducing neuroinflammation and mitigating cognitive decline [23]. Reduced mastication, such as through soft diets or dental issues, has been linked to increased risk of dementia and neurodegenerative conditions, demonstrating how oral activity directly influences cognitive function [3, 13]. However, it is clear from our results that increases in astrocyte complexity can also be associated with reduced cognitive performance following consumption of the soft diet, which highlights that it is unsafe to establish rules relating morphology to functional outcomes.

Recent studies have highlighted the complex relationship between astrocyte morphology, cognitive function, and dietary factors. While increased astrocyte complexity is often associated with improved cognitive performance, indeed, our previous has shown that a soft diet can lead to reduced cognitive abilities despite increased astrocyte complexity [14, 23]. High-fat diets have also been found to increase astrocyte size and complexity, enhancing glutamate clearance and synaptic plasticity [42]. However, the effects of diet on astrocytes vary across brain regions and can be influenced by factors such as age and environmental enrichment [43]. Thus, astrocyte responses to dietary changes and their impact on cognitive function are not straightforward, which emphasizes the need for further research to understand the role of astrocytes in various conditions and their molecular phenotype rather than making assumptions based on their morphological phenotype [44, 45]. Although astrocytes undergo complex morphological and functional changes with age, which may contribute to cognitive decline. In middle-aged mice, astrocytes have been found to show increased complexity in specific hippocampal regions associated with cognition [46]. This increased complexity is counterintuitive, as it occurs alongside neuronal loss and behavioral deficits [22, 23]. Aging astrocytes exhibit a reactive phenotype resembling neuroinflammatory A1-like astrocytes, potentially impairing their normal functions [39]. However, in models of Alzheimer-like neurodegeneration, astrocytes show early degeneration and atrophy, followed by reactivity around amyloid plaques [40], which highlights how the evolution of astrocyte morphology is more dynamic and, at the very least, non-linear.

In this context, future investigations evaluating astrocytic morphology and function in additional brain regions may help clarify whether the effects observed here represent a predominantly hippocampal phenomenon or part of a more widespread neural response to masticatory modulation across the lifespan.

Although our analyses focused on the hippocampus, previous studies such as those by Onozuka have demonstrated that masticatory activity recruits multiple brain regions, supporting the biological plausibility of broader neural effects [2]. Nevertheless, evidence regarding astrocytic remodeling associated with masticatory function remains more consistently described within hippocampal circuits, including the dentate gyrus, as previously reported by Siqueira-Mendes [14].

### 4.4. Spatial learning patterns in the context of astrocytic morphological heterogeneity

Interestingly, the dorsal SLM exhibited marked phenotypic heterogeneity across the experimental groups, particularly regarding the distribution of astrocytic complexity subtypes. This regional variability prompted us to revisit previously published behavioral findings from Siqueira-Mendes et al. [14] in order to qualitatively compare hippocampal morphometric patterns with spatial learning performance under different masticatory regimens.

In the behavioral dataset, aged animals subjected to the HD/SD condition did not differ from young animals in the latency contrast index on the fourth training day, whereas aged animals maintained under HD or rehabilitated through the HD/SD/HD regimen exhibited higher contrast index values. Although the present study was not designed to establish direct behavioral correlations, these findings are noteworthy because the dorsal hippocampal region, particularly the stratum lacunosum-moleculare, is critically involved in spatial information processing and hippocampal plasticity.

Therefore, the astrocytic heterogeneity observed in the dorsal SLM may reflect distinct patterns of structural remodeling associated with masticatory conditions and aging, which qualitatively parallel previously reported differences in spatial learning performance [6].

## Concluding remarks

Modern lifestyles have confined humans within a metaphorical “cage” that restricts neuroplasticity [47]. Similarly, the impoverished environments of standard laboratory cages, which simulate sedentary living, likely impair neuroplasticity in rodents [14, 22, 23, 37]. In both humans and rodents, sedentary behavior hides a complex interplay of molecular, cellular, and systemic factors that contribute to age-related cognitive decline [48]. To mitigate these effects, enriched environments (providing multisensory, motor, and cognitive stimuli) have been employed to enhance neuroplasticity and slow cognitive decline [49]. However, since all mice in the present study were housed in small groups within standard laboratory cages that simulate sedentary lifestyles, it is reasonable to assume that exposure to stimuli promoting neuroplasticity was limited. This controlled environment allowed us to isolate the specific effects of aging and masticatory activity on astrocytic plasticity. Given the critical role of astrocytes in supporting synaptic plasticity, masticatory activity emerges as a valuable non-pharmacological tool to preserve astrocytic integrity and reduce cognitive impairment. The study of astrocyte morphology within the lacunosum-molecular (LM) layer of the hippocampus provided critical insights into the cellular underpinnings of age-related cognitive decline. Aging induces profound changes in astrocytes, including reductions in complexity, branching, and synaptic coverage, compromising their ability to maintain synaptic plasticity, metabolic support, and neurotransmitter homeostasis [25]. These cellular alterations have systemic repercussions, contributing to chronic low-grade neuroinflammation, impaired memory consolidation, and spatial learning deficits—hallmarks of age-related cognitive impairments [39, 40].

The progressive decline in cognitive abilities, observed through the Morris Water Maze, is tightly coupled with astrocytic dysfunction in the hippocampus. As astrocytes lose their structural integrity, circuits involved in memory and learning, such as those in the temporoammonic pathway, become less effective. This impairs the ability of the hippocampus to integrate spatial and episodic memory, leading to decreased cognitive performance [50].

Masticatory activity is a valuable behavioral intervention that modulates these age-related cellular changes. Chewing stimulates sensory and neural pathways that activate hippocampal networks, promoting astrocytic complexity and delaying cognitive decline [1, 23]. This study demonstrates that dietary regimens incorporating hard and soft food mimic real-life scenarios of fluctuating masticatory function, revealing the potential for rehabilitation through lifestyle interventions.

An important limitation of the present study concerns the use of GFAP immunolabeling for astrocytic morphometric reconstruction. Although GFAP is widely used for the identification and structural analysis of astrocytes, this marker predominantly labels intermediate filaments of the astrocytic cytoskeleton and does not fully encompass the entire astrocytic territory, particularly the finest distal and highly ramified peripheral processes. Therefore, the present reconstructions primarily reflect remodeling of the main astrocytic structural framework rather than the complete cellular morphology. In addition, GFAP expression may vary according to brain region, physiological state, aging, and astrocytic reactivity. Consequently, some of the morphological differences observed across aging and masticatory conditions may also be influenced by distinct astroglial activation states. Nevertheless, GFAP-based three-dimensional reconstructions remain a well-established and reproducible approach for investigating structural astroglial remodeling, especially when associated with branching organization and complexity analyses. Future studies combining complementary astrocytic markers and higher-resolution approaches may further expand the understanding of the phenotype-specific astroglial adaptations observed in the present study.

In conclusion, the intricate relationship between astrocytic morphology, cognitive performance, and environmental factors underscores the critical need for early interventions to preserve cognitive resilience throughout aging. By establishing connections between cellular alterations and behavioral outcomes, this research highlights the significance of integrative strategies—such as dietary and lifestyle interventions—in supporting brain health across the lifespan. Notably, masticatory activity emerges as a critical factor in sustaining astrocytic integrity and should be incorporated into non-pharmacological approaches to mitigate age-related cognitive decline and enhance neuroplasticity.

## Supplementary Information

Below is the link to the electronic supplementary material.

Supplementary Material 1.

Supplementary Material 2.

Supplementary Material 3.

Supplementary Material 4.

Supplementary Material 5 (Supplementary Fig. 1. Region of interest (ROI) in the CA1 stratum lacunosum-moleculare (SLM) used for astrocyte reconstruction. The ROI is outlined in yellow, while a systematic sampling grid (120 × 120 µm; orange) containing reconstruction frames (50 × 50 µm; green) was applied throughout the region for astrocyte selection and analysis.).

## Data Availability

No datasets were generated or analysed during the current study.
