# Supplementary material for: The role of astrocytes in the temporoammonic pathway: masticatory behavior as a neuroprotective strategy against age-related cognitive decline
Source: Behav Brain Funct. 2026 Jul 28;22:51. doi: 10.1186/s12993-026-00358-6 (PMC13700188; doi:10.1186/s12993-026-00358-6)
Supplement: Supplementary file 1 [file 12993_2026_358_MOESM1_ESM.pdf]

2way ANOVA
ANOVA results

1	Table Analyzed	TESTE ANOVA - HD				
2						
3	**Two-way ANOVA**	Ordinary				
4	Alpha	0.05				
5						
6	**Source of Variation**	**% of total variation**	**P value**	**P value summary**	**Significant?**	
7	Interaction	1.949	0.6552	ns	No	
8	Ages	49.36	<0.0001	****	Yes	
9	Days	10.54	0.0476	*	Yes	
10						
11	**ANOVA table**	**SS**	**DF**	**MS**	**F (DFn, DFd)**	**P value**
12	Interaction	0.03718	3	0.01239	F (3, 32) = 0.5448	P=0.6552
13	Ages	0.9419	1	0.9419	F (1, 32) = 41.41	P<0.0001
14	Days	0.2012	3	0.06706	F (3, 32) = 2.948	P=0.0476
15	Residual	0.7279	32	0.02275		
16						
17	**Difference between row means**					
18	Mean of 6M	0.4102				
19	Mean of 18M	0.1033				
20	Difference between means	0.3069				
21	SE of difference	0.04769				
22	95% CI of difference	0.2098 to 0.4040				
23						
24	**Data summary**					
25	Number of columns (Days)	4				
26	Number of rows (Ages)	2				
27	Number of values	40				

2way ANOVA
Multiple comparisons

1	Compare cell means regardless of rows and column							
2								
3	Number of families	1						
4	Number of comparisons per family	28						
5	Alpha	0.05						
6								
7	**Tukey's multiple comparisons test**	**Mean Diff.**	**95.00% CI of diff.**	**Below threshold?**	**Summary**	**Adjusted P Value**		
8								
9	6M:HD - D1 vs D2 vs. 6M:HD - D1 vs D3	-0.07960	-0.3886 to 0.2294	No	ns	0.9896		
10	6M:HD - D1 vs D2 vs. 6M:HD - D1 vs D4	-0.2626	-0.5716 to 0.04639	No	ns	0.1436		
11	6M:HD - D1 vs D2 vs. 6M:HD - D1 vs D5	-0.07460	-0.3836 to 0.2344	No	ns	0.9929		
12	6M:HD - D1 vs D2 vs. 18M:HD - D1 vs D2	0.2858	-0.02319 to 0.5948	No	ns	0.0864		
13	6M:HD - D1 vs D2 vs. 18M:HD - D1 vs D3	0.1894	-0.1196 to 0.4984	No	ns	0.5065		
14	6M:HD - D1 vs D2 vs. 18M:HD - D1 vs D4	0.1488	-0.1602 to 0.4578	No	ns	0.7695		
15	6M:HD - D1 vs D2 vs. 18M:HD - D1 vs D5	0.1868	-0.1222 to 0.4958	No	ns	0.5237		
16	6M:HD - D1 vs D3 vs. 6M:HD - D1 vs D4	-0.1830	-0.4920 to 0.1260	No	ns	0.5490		
17	6M:HD - D1 vs D3 vs. 6M:HD - D1 vs D5	0.005000	-0.3040 to 0.3140	No	ns	>0.9999		
18	6M:HD - D1 vs D3 vs. 18M:HD - D1 vs D2	0.3654	0.05641 to 0.6744	Yes	*	0.0116		
19	6M:HD - D1 vs D3 vs. 18M:HD - D1 vs D3	0.2690	-0.03999 to 0.5780	No	ns	0.1253		
20	6M:HD - D1 vs D3 vs. 18M:HD - D1 vs D4	0.2284	-0.08059 to 0.5374	No	ns	0.2781		
21	6M:HD - D1 vs D3 vs. 18M:HD - D1 vs D5	0.2664	-0.04259 to 0.5754	No	ns	0.1325		
22	6M:HD - D1 vs D4 vs. 6M:HD - D1 vs D5	0.1880	-0.1210 to 0.4970	No	ns	0.5158		
23	6M:HD - D1 vs D4 vs. 18M:HD - D1 vs D2	0.5484	0.2394 to 0.8574	Yes	****	<0.0001		
24	6M:HD - D1 vs D4 vs. 18M:HD - D1 vs D3	0.4520	0.1430 to 0.7610	Yes	***	0.0010		
25	6M:HD - D1 vs D4 vs. 18M:HD - D1 vs D4	0.4114	0.1024 to 0.7204	Yes	**	0.0032		
26	6M:HD - D1 vs D4 vs. 18M:HD - D1 vs D5	0.4494	0.1404 to 0.7584	Yes	**	0.0011		
27	6M:HD - D1 vs D5 vs. 18M:HD - D1 vs D2	0.3604	0.05141 to 0.6694	Yes	*	0.0133		
28	6M:HD - D1 vs D5 vs. 18M:HD - D1 vs D3	0.2640	-0.04499 to 0.5730	No	ns	0.1394		
29	6M:HD - D1 vs D5 vs. 18M:HD - D1 vs D4	0.2234	-0.08559 to 0.5324	No	ns	0.3033		
30	6M:HD - D1 vs D5 vs. 18M:HD - D1 vs D5	0.2614	-0.04759 to 0.5704	No	ns	0.1472		

	**2way ANOVA** Multiple comparisons							
31	18M:HD - D1 vs D2 vs. 18M:HD - D1 vs D3	-0.09640	-0.4054 to 0.2126	No	ns	0.9694		
32	18M:HD - D1 vs D2 vs. 18M:HD - D1 vs D4	-0.1370	-0.4460 to 0.1720	No	ns	0.8337		
33	18M:HD - D1 vs D2 vs. 18M:HD - D1 vs D5	-0.09900	-0.4080 to 0.2100	No	ns	0.9647		
34	18M:HD - D1 vs D3 vs. 18M:HD - D1 vs D4	-0.04060	-0.3496 to 0.2684	No	ns	0.9999		
35	18M:HD - D1 vs D3 vs. 18M:HD - D1 vs D5	-0.002600	-0.3116 to 0.3064	No	ns	>0.9999		
36	18M:HD - D1 vs D4 vs. 18M:HD - D1 vs D5	0.03800	-0.2710 to 0.3470	No	ns	>0.9999		
37								
38								
39	**Test details**	**Mean 1**	**Mean 2**	**Mean Diff.**	**SE of diff.**	**N1**	**N2**	**q**
40								
41	6M:HD - D1 vs D2 vs. 6M:HD - D1 vs D3	0.3060	0.3856	-0.07960	0.09539	5	5	1.180
42	6M:HD - D1 vs D2 vs. 6M:HD - D1 vs D4	0.3060	0.5686	-0.2626	0.09539	5	5	3.893
43	6M:HD - D1 vs D2 vs. 6M:HD - D1 vs D5	0.3060	0.3806	-0.07460	0.09539	5	5	1.106
44	6M:HD - D1 vs D2 vs. 18M:HD - D1 vs D2	0.3060	0.02020	0.2858	0.09539	5	5	4.237
45	6M:HD - D1 vs D2 vs. 18M:HD - D1 vs D3	0.3060	0.1166	0.1894	0.09539	5	5	2.808
46	6M:HD - D1 vs D2 vs. 18M:HD - D1 vs D4	0.3060	0.1572	0.1488	0.09539	5	5	2.206
47	6M:HD - D1 vs D2 vs. 18M:HD - D1 vs D5	0.3060	0.1192	0.1868	0.09539	5	5	2.769
48	6M:HD - D1 vs D3 vs. 6M:HD - D1 vs D4	0.3856	0.5686	-0.1830	0.09539	5	5	2.713
49	6M:HD - D1 vs D3 vs. 6M:HD - D1 vs D5	0.3856	0.3806	0.005000	0.09539	5	5	0.07413
50	6M:HD - D1 vs D3 vs. 18M:HD - D1 vs D2	0.3856	0.02020	0.3654	0.09539	5	5	5.417
51	6M:HD - D1 vs D3 vs. 18M:HD - D1 vs D3	0.3856	0.1166	0.2690	0.09539	5	5	3.988
52	6M:HD - D1 vs D3 vs. 18M:HD - D1 vs D4	0.3856	0.1572	0.2284	0.09539	5	5	3.386
53	6M:HD - D1 vs D3 vs. 18M:HD - D1 vs D5	0.3856	0.1192	0.2664	0.09539	5	5	3.950
54	6M:HD - D1 vs D4 vs. 6M:HD - D1 vs D5	0.5686	0.3806	0.1880	0.09539	5	5	2.787
55	6M:HD - D1 vs D4 vs. 18M:HD - D1 vs D2	0.5686	0.02020	0.5484	0.09539	5	5	8.131
56	6M:HD - D1 vs D4 vs. 18M:HD - D1 vs D3	0.5686	0.1166	0.4520	0.09539	5	5	6.701
57	6M:HD - D1 vs D4 vs. 18M:HD - D1 vs D4	0.5686	0.1572	0.4114	0.09539	5	5	6.099
58	6M:HD - D1 vs D4 vs. 18M:HD - D1 vs D5	0.5686	0.1192	0.4494	0.09539	5	5	6.663
59	6M:HD - D1 vs D5 vs. 18M:HD - D1 vs D2	0.3806	0.02020	0.3604	0.09539	5	5	5.343
60	6M:HD - D1 vs D5 vs. 18M:HD - D1 vs D3	0.3806	0.1166	0.2640	0.09539	5	5	3.914

**2way ANOVA**<br>Multiple comparisons								
61	6M:HD - D1 vs D5 vs. 18M:HD - D1 vs D4	0.3806	0.1572	0.2234	0.09539	5	5	3.312
62	6M:HD - D1 vs D5 vs. 18M:HD - D1 vs D5	0.3806	0.1192	0.2614	0.09539	5	5	3.875
63	18M:HD - D1 vs D2 vs. 18M:HD - D1 vs D3	0.02020	0.1166	-0.09640	0.09539	5	5	1.429
64	18M:HD - D1 vs D2 vs. 18M:HD - D1 vs D4	0.02020	0.1572	-0.1370	0.09539	5	5	2.031
65	18M:HD - D1 vs D2 vs. 18M:HD - D1 vs D5	0.02020	0.1192	-0.09900	0.09539	5	5	1.468
66	18M:HD - D1 vs D3 vs. 18M:HD - D1 vs D4	0.1166	0.1572	-0.04060	0.09539	5	5	0.6019
67	18M:HD - D1 vs D3 vs. 18M:HD - D1 vs D5	0.1166	0.1192	-0.002600	0.09539	5	5	0.03855
68	18M:HD - D1 vs D4 vs. 18M:HD - D1 vs D5	0.1572	0.1192	0.03800	0.09539	5	5	0.5634

1	
2	
3	
4	
5	
6	
7	
8	
9	
10	
11	
12	
13	
14	
15	
16	
17	
18	
19	
20	
21	
22	
23	
24	
25	
26	
27	
28	
29	
30	

31	
32	
33	
34	
35	
36	
37	
38	
39	**DF**
40	
41	32.00
42	32.00
43	32.00
44	32.00
45	32.00
46	32.00
47	32.00
48	32.00
49	32.00
50	32.00
51	32.00
52	32.00
53	32.00
54	32.00
55	32.00
56	32.00
57	32.00
58	32.00
59	32.00
60	32.00

61	32.00
62	32.00
63	32.00
64	32.00
65	32.00
66	32.00
67	32.00
68	32.00
