# Supplementary material for: The role of astrocytes in the temporoammonic pathway: masticatory behavior as a neuroprotective strategy against age-related cognitive decline
Source: Behav Brain Funct. 2026 Jul 28;22:51. doi: 10.1186/s12993-026-00358-6 (PMC13700188; doi:10.1186/s12993-026-00358-6)
Supplement: Supplementary file 2 [file 12993_2026_358_MOESM2_ESM.pdf]

2way ANOVA<br>ANOVA results						
1	Table Analyzed	TESTE ANOVA - HD/SD/HD				
2						
3	**Two-way ANOVA**	Ordinary				
4	Alpha	0.05				
5						
6	**Source of Variation**	**% of total variation**	**P value**	**P value summary**	**Significant?**	
7	Interaction	0.8512	0.9381	ns	No	
8	Ages	9.605	0.0399	*	Yes	
9	Days	22.57	0.0241	*	Yes	
10						
11	**ANOVA table**	**SS**	**DF**	**MS**	**F (DFn, DFd)**	**P value**
12	Interaction	0.01889	3	0.006296	F (3, 32) = 0.1356	P=0.9381
13	Ages	0.2132	1	0.2132	F (1, 32) = 4.589	P=0.0399
14	Days	0.5008	3	0.1669	F (3, 32) = 3.594	P=0.0241
15	Residual	1.486	32	0.04645		
16						
17	**Difference between row means**					
18	Mean of 6M	0.2902				
19	Mean of 18M	0.1442				
20	Difference between means	0.1460				
21	SE of difference	0.06815				
22	95% CI of difference	0.007175 to 0.2848				
23						
24	**Data summary**					
25	Number of columns (Days)	4				
26	Number of rows (Ages)	2				
27	Number of values	40				

2way ANOVA<br>Multiple comparisons								
1	Compare cell means regardless of rows and columns							
2								
3	Number of families	1						
4	Number of comparisons per family	28						
5	Alpha	0.05						
6								
7	**Tukey's multiple comparisons test**	**Mean Diff.**	**95.00% CI of diff.**	**Below threshold?**	**Summary**	**Adjusted P Value**		
8								
9	6M:HD - D1 vs D2 vs. 6M:HD - D1 vs D3	0.01140	-0.4301 to 0.4529	No	ns	>0.9999		
10	6M:HD - D1 vs D2 vs. 6M:HD - D1 vs D4	-0.3090	-0.7505 to 0.1325	No	ns	0.3417		
11	6M:HD - D1 vs D2 vs. 6M:HD - D1 vs D5	-0.1208	-0.5623 to 0.3207	No	ns	0.9853		
12	6M:HD - D1 vs D2 vs. 18M:HD - D1 vs D2	0.1556	-0.2859 to 0.5971	No	ns	0.9422		
13	6M:HD - D1 vs D2 vs. 18M:HD - D1 vs D3	0.1000	-0.3415 to 0.5415	No	ns	0.9952		
14	6M:HD - D1 vs D2 vs. 18M:HD - D1 vs D4	-0.1002	-0.5417 to 0.3413	No	ns	0.9951		
15	6M:HD - D1 vs D2 vs. 18M:HD - D1 vs D5	0.01020	-0.4313 to 0.4517	No	ns	>0.9999		
16	6M:HD - D1 vs D3 vs. 6M:HD - D1 vs D4	-0.3204	-0.7619 to 0.1211	No	ns	0.2991		
17	6M:HD - D1 vs D3 vs. 6M:HD - D1 vs D5	-0.1322	-0.5737 to 0.3093	No	ns	0.9756		
18	6M:HD - D1 vs D3 vs. 18M:HD - D1 vs D2	0.1442	-0.2973 to 0.5857	No	ns	0.9609		
19	6M:HD - D1 vs D3 vs. 18M:HD - D1 vs D3	0.08860	-0.3529 to 0.5301	No	ns	0.9977		
20	6M:HD - D1 vs D3 vs. 18M:HD - D1 vs D4	-0.1116	-0.5531 to 0.3299	No	ns	0.9907		
21	6M:HD - D1 vs D3 vs. 18M:HD - D1 vs D5	-0.001200	-0.4427 to 0.4403	No	ns	>0.9999		
22	6M:HD - D1 vs D4 vs. 6M:HD - D1 vs D5	0.1882	-0.2533 to 0.6297	No	ns	0.8592		
23	6M:HD - D1 vs D4 vs. 18M:HD - D1 vs D2	0.4646	0.02306 to 0.9061	Yes	*	0.0335		
24	6M:HD - D1 vs D4 vs. 18M:HD - D1 vs D3	0.4090	-0.03254 to 0.8505	No	ns	0.0856		
25	6M:HD - D1 vs D4 vs. 18M:HD - D1 vs D4	0.2088	-0.2327 to 0.6503	No	ns	0.7849		
26	6M:HD - D1 vs D4 vs. 18M:HD - D1 vs D5	0.3192	-0.1223 to 0.7607	No	ns	0.3034		
27	6M:HD - D1 vs D5 vs. 18M:HD - D1 vs D2	0.2764	-0.1651 to 0.7179	No	ns	0.4802		
28	6M:HD - D1 vs D5 vs. 18M:HD - D1 vs D3	0.2208	-0.2207 to 0.6623	No	ns	0.7353		
29	6M:HD - D1 vs D5 vs. 18M:HD - D1 vs D4	0.02060	-0.4209 to 0.4621	No	ns	>0.9999		
30	6M:HD - D1 vs D5 vs. 18M:HD - D1 vs D5	0.1310	-0.3105 to 0.5725	No	ns	0.9768		

**2way ANOVA**<br>Multiple comparisons								
31	18M:HD - D1 vs D2 vs. 18M:HD - D1 vs D3	-0.05560	-0.4971 to 0.3859	No	ns	0.9999		
32	18M:HD - D1 vs D2 vs. 18M:HD - D1 vs D4	-0.2558	-0.6973 to 0.1857	No	ns	0.5757		
33	18M:HD - D1 vs D2 vs. 18M:HD - D1 vs D5	-0.1454	-0.5869 to 0.2961	No	ns	0.9592		
34	18M:HD - D1 vs D3 vs. 18M:HD - D1 vs D4	-0.2002	-0.6417 to 0.2413	No	ns	0.8177		
35	18M:HD - D1 vs D3 vs. 18M:HD - D1 vs D5	-0.08980	-0.5313 to 0.3517	No	ns	0.9975		
36	18M:HD - D1 vs D4 vs. 18M:HD - D1 vs D5	0.1104	-0.3311 to 0.5519	No	ns	0.9913		
37								
38								
39	**Test details**	**Mean 1**	**Mean 2**	**Mean Diff.**	**SE of diff.**	**N1**	**N2**	**q**
40								
41	6M:HD - D1 vs D2 vs. 6M:HD - D1 vs D3	0.1856	0.1742	0.01140	0.1363	5	5	0.1183
42	6M:HD - D1 vs D2 vs. 6M:HD - D1 vs D4	0.1856	0.4946	-0.3090	0.1363	5	5	3.206
43	6M:HD - D1 vs D2 vs. 6M:HD - D1 vs D5	0.1856	0.3064	-0.1208	0.1363	5	5	1.253
44	6M:HD - D1 vs D2 vs. 18M:HD - D1 vs D2	0.1856	0.03000	0.1556	0.1363	5	5	1.614
45	6M:HD - D1 vs D2 vs. 18M:HD - D1 vs D3	0.1856	0.08560	0.1000	0.1363	5	5	1.038
46	6M:HD - D1 vs D2 vs. 18M:HD - D1 vs D4	0.1856	0.2858	-0.1002	0.1363	5	5	1.040
47	6M:HD - D1 vs D2 vs. 18M:HD - D1 vs D5	0.1856	0.1754	0.01020	0.1363	5	5	0.1058
48	6M:HD - D1 vs D3 vs. 6M:HD - D1 vs D4	0.1742	0.4946	-0.3204	0.1363	5	5	3.324
49	6M:HD - D1 vs D3 vs. 6M:HD - D1 vs D5	0.1742	0.3064	-0.1322	0.1363	5	5	1.372
50	6M:HD - D1 vs D3 vs. 18M:HD - D1 vs D2	0.1742	0.03000	0.1442	0.1363	5	5	1.496
51	6M:HD - D1 vs D3 vs. 18M:HD - D1 vs D3	0.1742	0.08560	0.08860	0.1363	5	5	0.9192
52	6M:HD - D1 vs D3 vs. 18M:HD - D1 vs D4	0.1742	0.2858	-0.1116	0.1363	5	5	1.158
53	6M:HD - D1 vs D3 vs. 18M:HD - D1 vs D5	0.1742	0.1754	-0.001200	0.1363	5	5	0.01245
54	6M:HD - D1 vs D4 vs. 6M:HD - D1 vs D5	0.4946	0.3064	0.1882	0.1363	5	5	1.953
55	6M:HD - D1 vs D4 vs. 18M:HD - D1 vs D2	0.4946	0.03000	0.4646	0.1363	5	5	4.820
56	6M:HD - D1 vs D4 vs. 18M:HD - D1 vs D3	0.4946	0.08560	0.4090	0.1363	5	5	4.243
57	6M:HD - D1 vs D4 vs. 18M:HD - D1 vs D4	0.4946	0.2858	0.2088	0.1363	5	5	2.166
58	6M:HD - D1 vs D4 vs. 18M:HD - D1 vs D5	0.4946	0.1754	0.3192	0.1363	5	5	3.312
59	6M:HD - D1 vs D5 vs. 18M:HD - D1 vs D2	0.3064	0.03000	0.2764	0.1363	5	5	2.868
60	6M:HD - D1 vs D5 vs. 18M:HD - D1 vs D3	0.3064	0.08560	0.2208	0.1363	5	5	2.291

**2way ANOVA**<br>Multiple comparisons								
61	6M:HD - D1 vs D5 vs. 18M:HD - D1 vs D4	0.3064	0.2858	0.02060	0.1363	5	5	0.2137
62	6M:HD - D1 vs D5 vs. 18M:HD - D1 vs D5	0.3064	0.1754	0.1310	0.1363	5	5	1.359
63	18M:HD - D1 vs D2 vs. 18M:HD - D1 vs D3	0.03000	0.08560	-0.05560	0.1363	5	5	0.5769
64	18M:HD - D1 vs D2 vs. 18M:HD - D1 vs D4	0.03000	0.2858	-0.2558	0.1363	5	5	2.654
65	18M:HD - D1 vs D2 vs. 18M:HD - D1 vs D5	0.03000	0.1754	-0.1454	0.1363	5	5	1.509
66	18M:HD - D1 vs D3 vs. 18M:HD - D1 vs D4	0.08560	0.2858	-0.2002	0.1363	5	5	2.077
67	18M:HD - D1 vs D3 vs. 18M:HD - D1 vs D5	0.08560	0.1754	-0.08980	0.1363	5	5	0.9317
68	18M:HD - D1 vs D4 vs. 18M:HD - D1 vs D5	0.2858	0.1754	0.1104	0.1363	5	5	1.145

1	
2	
3	
4	
5	
6	
7	
8	
9	
10	
11	
12	
13	
14	
15	
16	
17	
18	
19	
20	
21	
22	
23	
24	
25	
26	
27	
28	
29	
30	

31	
32	
33	
34	
35	
36	
37	
38	
39	**DF**
40	
41	32.00
42	32.00
43	32.00
44	32.00
45	32.00
46	32.00
47	32.00
48	32.00
49	32.00
50	32.00
51	32.00
52	32.00
53	32.00
54	32.00
55	32.00
56	32.00
57	32.00
58	32.00
59	32.00
60	32.00

61	32.00
62	32.00
63	32.00
64	32.00
65	32.00
66	32.00
67	32.00
68	32.00
