# Supplementary material for: The role of astrocytes in the temporoammonic pathway: masticatory behavior as a neuroprotective strategy against age-related cognitive decline
Source: Behav Brain Funct. 2026 Jul 28;22:51. doi: 10.1186/s12993-026-00358-6 (PMC13700188; doi:10.1186/s12993-026-00358-6)
Supplement: Supplementary file 3 [file 12993_2026_358_MOESM3_ESM.pdf]

2way ANOVA<br>ANOVA results						
1	Table Analyzed	TESTE ANOVA - HD/SD				
2						
3	**Two-way ANOVA**	Ordinary				
4	Alpha	0.05				
5						
6	**Source of Variation**	**% of total variation**	**P value**	**P value summary**	**Significant?**	
7	Interaction	0.6947	0.9688	ns	No	
8	Ages	0.03617	0.9101	ns	No	
9	Days	9.911	0.3316	ns	No	
10						
11	**ANOVA table**	**SS**	**DF**	**MS**	**F (DFn, DFd)**	**P value**
12	Interaction	0.006350	3	0.002117	F (3, 32) = 0.08293	P=0.9688
13	Ages	0.0003306	1	0.0003306	F (1, 32) = 0.01295	P=0.9101
14	Days	0.09059	3	0.03020	F (3, 32) = 1.183	P=0.3316
15	Residual	0.8168	32	0.02552		
16						
17	**Difference between row means**					
18	Mean of 6M	0.2023				
19	Mean of 18M	0.2081				
20	Difference between means	-0.005750				
21	SE of difference	0.05052				
22	95% CI of difference	-0.1087 to 0.09716				
23						
24	**Data summary**					
25	Number of columns (Days)	4				
26	Number of rows (Ages)	2				
27	Number of values	40				

2way ANOVA<br>Multiple comparisons								
1	Compare cell means regardless of rows and columns							
2								
3	Number of families	1						
4	Number of comparisons per family	28						
5	Alpha	0.05						
6								
7	**Tukey's multiple comparisons test**	**Mean Diff.**	**95.00% CI of diff.**	**Below threshold?**	**Summary**	**Adjusted P Value**		
8								
9	6M:HD - D1 vs D2 vs. 6M:HD - D1 vs D3	-0.07040	-0.3977 to 0.2569	No	ns	0.9965		
10	6M:HD - D1 vs D2 vs. 6M:HD - D1 vs D4	-0.1544	-0.4817 to 0.1729	No	ns	0.7869		
11	6M:HD - D1 vs D2 vs. 6M:HD - D1 vs D5	-0.09640	-0.4237 to 0.2309	No	ns	0.9777		
12	6M:HD - D1 vs D2 vs. 18M:HD - D1 vs D2	-0.02020	-0.3475 to 0.3071	No	ns	>0.9999		
13	6M:HD - D1 vs D2 vs. 18M:HD - D1 vs D3	-0.07880	-0.4061 to 0.2485	No	ns	0.9931		
14	6M:HD - D1 vs D2 vs. 18M:HD - D1 vs D4	-0.1186	-0.4459 to 0.2087	No	ns	0.9336		
15	6M:HD - D1 vs D2 vs. 18M:HD - D1 vs D5	-0.1266	-0.4539 to 0.2007	No	ns	0.9091		
16	6M:HD - D1 vs D3 vs. 6M:HD - D1 vs D4	-0.08400	-0.4113 to 0.2433	No	ns	0.9898		
17	6M:HD - D1 vs D3 vs. 6M:HD - D1 vs D5	-0.02600	-0.3533 to 0.3013	No	ns	>0.9999		
18	6M:HD - D1 vs D3 vs. 18M:HD - D1 vs D2	0.05020	-0.2771 to 0.3775	No	ns	0.9996		
19	6M:HD - D1 vs D3 vs. 18M:HD - D1 vs D3	-0.008400	-0.3357 to 0.3189	No	ns	>0.9999		
20	6M:HD - D1 vs D3 vs. 18M:HD - D1 vs D4	-0.04820	-0.3755 to 0.2791	No	ns	0.9997		
21	6M:HD - D1 vs D3 vs. 18M:HD - D1 vs D5	-0.05620	-0.3835 to 0.2711	No	ns	0.9992		
22	6M:HD - D1 vs D4 vs. 6M:HD - D1 vs D5	0.05800	-0.2693 to 0.3853	No	ns	0.9990		
23	6M:HD - D1 vs D4 vs. 18M:HD - D1 vs D2	0.1342	-0.1931 to 0.4615	No	ns	0.8812		
24	6M:HD - D1 vs D4 vs. 18M:HD - D1 vs D3	0.07560	-0.2517 to 0.4029	No	ns	0.9946		
25	6M:HD - D1 vs D4 vs. 18M:HD - D1 vs D4	0.03580	-0.2915 to 0.3631	No	ns	>0.9999		
26	6M:HD - D1 vs D4 vs. 18M:HD - D1 vs D5	0.02780	-0.2995 to 0.3551	No	ns	>0.9999		
27	6M:HD - D1 vs D5 vs. 18M:HD - D1 vs D2	0.07620	-0.2511 to 0.4035	No	ns	0.9943		
28	6M:HD - D1 vs D5 vs. 18M:HD - D1 vs D3	0.01760	-0.3097 to 0.3449	No	ns	>0.9999		
29	6M:HD - D1 vs D5 vs. 18M:HD - D1 vs D4	-0.02220	-0.3495 to 0.3051	No	ns	>0.9999		
30	6M:HD - D1 vs D5 vs. 18M:HD - D1 vs D5	-0.03020	-0.3575 to 0.2971	No	ns	>0.9999		

2way ANOVA<br>Multiple comparisons								
31	18M:HD - D1 vs D2 vs. 18M:HD - D1 vs D3	-0.05860	-0.3859 to 0.2687	No	ns	0.9989		
32	18M:HD - D1 vs D2 vs. 18M:HD - D1 vs D4	-0.09840	-0.4257 to 0.2289	No	ns	0.9750		
33	18M:HD - D1 vs D2 vs. 18M:HD - D1 vs D5	-0.1064	-0.4337 to 0.2209	No	ns	0.9619		
34	18M:HD - D1 vs D3 vs. 18M:HD - D1 vs D4	-0.03980	-0.3671 to 0.2875	No	ns	>0.9999		
35	18M:HD - D1 vs D3 vs. 18M:HD - D1 vs D5	-0.04780	-0.3751 to 0.2795	No	ns	0.9997		
36	18M:HD - D1 vs D4 vs. 18M:HD - D1 vs D5	-0.008000	-0.3353 to 0.3193	No	ns	>0.9999		
37								
38								
39	**Test details**	**Mean 1**	**Mean 2**	**Mean Diff.**	**SE of diff.**	**N1**	**N2**	**q**
40								
41	6M:HD - D1 vs D2 vs. 6M:HD - D1 vs D3	0.1220	0.1924	-0.07040	0.1010	5	5	0.9853
42	6M:HD - D1 vs D2 vs. 6M:HD - D1 vs D4	0.1220	0.2764	-0.1544	0.1010	5	5	2.161
43	6M:HD - D1 vs D2 vs. 6M:HD - D1 vs D5	0.1220	0.2184	-0.09640	0.1010	5	5	1.349
44	6M:HD - D1 vs D2 vs. 18M:HD - D1 vs D2	0.1220	0.1422	-0.02020	0.1010	5	5	0.2827
45	6M:HD - D1 vs D2 vs. 18M:HD - D1 vs D3	0.1220	0.2008	-0.07880	0.1010	5	5	1.103
46	6M:HD - D1 vs D2 vs. 18M:HD - D1 vs D4	0.1220	0.2406	-0.1186	0.1010	5	5	1.660
47	6M:HD - D1 vs D2 vs. 18M:HD - D1 vs D5	0.1220	0.2486	-0.1266	0.1010	5	5	1.772
48	6M:HD - D1 vs D3 vs. 6M:HD - D1 vs D4	0.1924	0.2764	-0.08400	0.1010	5	5	1.176
49	6M:HD - D1 vs D3 vs. 6M:HD - D1 vs D5	0.1924	0.2184	-0.02600	0.1010	5	5	0.3639
50	6M:HD - D1 vs D3 vs. 18M:HD - D1 vs D2	0.1924	0.1422	0.05020	0.1010	5	5	0.7026
51	6M:HD - D1 vs D3 vs. 18M:HD - D1 vs D3	0.1924	0.2008	-0.008400	0.1010	5	5	0.1176
52	6M:HD - D1 vs D3 vs. 18M:HD - D1 vs D4	0.1924	0.2406	-0.04820	0.1010	5	5	0.6746
53	6M:HD - D1 vs D3 vs. 18M:HD - D1 vs D5	0.1924	0.2486	-0.05620	0.1010	5	5	0.7866
54	6M:HD - D1 vs D4 vs. 6M:HD - D1 vs D5	0.2764	0.2184	0.05800	0.1010	5	5	0.8118
55	6M:HD - D1 vs D4 vs. 18M:HD - D1 vs D2	0.2764	0.1422	0.1342	0.1010	5	5	1.878
56	6M:HD - D1 vs D4 vs. 18M:HD - D1 vs D3	0.2764	0.2008	0.07560	0.1010	5	5	1.058
57	6M:HD - D1 vs D4 vs. 18M:HD - D1 vs D4	0.2764	0.2406	0.03580	0.1010	5	5	0.5011
58	6M:HD - D1 vs D4 vs. 18M:HD - D1 vs D5	0.2764	0.2486	0.02780	0.1010	5	5	0.3891
59	6M:HD - D1 vs D5 vs. 18M:HD - D1 vs D2	0.2184	0.1422	0.07620	0.1010	5	5	1.067
60	6M:HD - D1 vs D5 vs. 18M:HD - D1 vs D3	0.2184	0.2008	0.01760	0.1010	5	5	0.2463

**2way ANOVA**<br>Multiple comparisons								
61	6M:HD - D1 vs D5 vs. 18M:HD - D1 vs D4	0.2184	0.2406	-0.02220	0.1010	5	5	0.3107
62	6M:HD - D1 vs D5 vs. 18M:HD - D1 vs D5	0.2184	0.2486	-0.03020	0.1010	5	5	0.4227
63	18M:HD - D1 vs D2 vs. 18M:HD - D1 vs D3	0.1422	0.2008	-0.05860	0.1010	5	5	0.8202
64	18M:HD - D1 vs D2 vs. 18M:HD - D1 vs D4	0.1422	0.2406	-0.09840	0.1010	5	5	1.377
65	18M:HD - D1 vs D2 vs. 18M:HD - D1 vs D5	0.1422	0.2486	-0.1064	0.1010	5	5	1.489
66	18M:HD - D1 vs D3 vs. 18M:HD - D1 vs D4	0.2008	0.2406	-0.03980	0.1010	5	5	0.5571
67	18M:HD - D1 vs D3 vs. 18M:HD - D1 vs D5	0.2008	0.2486	-0.04780	0.1010	5	5	0.6690
68	18M:HD - D1 vs D4 vs. 18M:HD - D1 vs D5	0.2406	0.2486	-0.008000	0.1010	5	5	0.1120

1	
2	
3	
4	
5	
6	
7	
8	
9	
10	
11	
12	
13	
14	
15	
16	
17	
18	
19	
20	
21	
22	
23	
24	
25	
26	
27	
28	
29	
30	

31	
32	
33	
34	
35	
36	
37	
38	
39	**DF**
40	
41	32.00
42	32.00
43	32.00
44	32.00
45	32.00
46	32.00
47	32.00
48	32.00
49	32.00
50	32.00
51	32.00
52	32.00
53	32.00
54	32.00
55	32.00
56	32.00
57	32.00
58	32.00
59	32.00
60	32.00

61	32.00
62	32.00
63	32.00
64	32.00
65	32.00
66	32.00
67	32.00
68	32.00
