# Supplementary material for: The role of astrocytes in the temporoammonic pathway: masticatory behavior as a neuroprotective strategy against age-related cognitive decline
Source: Behav Brain Funct. 2026 Jul 28;22:51. doi: 10.1186/s12993-026-00358-6 (PMC13700188; doi:10.1186/s12993-026-00358-6)
Supplement: Supplementary file 4 [file 12993_2026_358_MOESM4_ESM.docx]

**Supplementary Table 1.** Morphometric features of astrocytes. Adapted from Carvalho-Paulo 2019.

| ***Branched Structure Analysis*** | |
| --- | --- |
| Segment | Any portion of astrocytes branched structure with endings that are either nodes or terminations with no intermediate nodes. |
| Segments/mm | Number of segments/total length of the segments expressed in millimeters. |
| Nº of Trees | Number of trees (dendritic segments per branch order) in the astrocytes |
| Total Nº of Segments | Refer to the total number of segments in the tree. |
| Single Branch Length | Total length of the line segments used to trace the branch of interest. |
| Mean Branch Length (µm) | Mean = [Total Length] / [Number of branches] |
| Total Branch Length (µm) | Total length for all branches in the tree. |
| Tortuosity | = [Actual length of the segment]/[Distance between the endpoints of the segment]. The smallest value is 1; this represents a straight segment. Tortuosity allows segments of different lengths to be compared in terms of the complexity of the paths they take. |
| Mean Branch Surface Area (µm²) | Computed by modeling each branch as a frustum (truncated right circular cone) divided by the number of branches. |
| Total Tree Surface Area (µm²) | 2D surface area of an astrocyte arbor computed based on the area defined by the endpoints of all trees. |
| Branch Volume (µm³) | Computed by modeling each piece of each branch as a frustum. |
| Total Branch Volume | Total volume for all branches in the tree. |
| Base Diameter of Primary Branch (µm) | Diameter at the start of the 1^st^ segment. |
| Planar Angle | Computed based on the endpoints of the segments. It refers to the change in direction of a segment relative to the previous segment. |
| Fractal Dimension  K-Dim | the “k-dim” of the fractal analysis, describes how the structure of interest fills space. Significant statistical differences in K-dim suggest morphological dissimilarities. |
| Convex Hull - Perimeter (µm), Area (µm²) 2D, Surface Area (µm²) 3D or Volume (µm³) | Convex hull measures the size of the branching field by interpreting a branched structure as a solid object controlling a given amount of physical space. The amount of physical space is defined in terms of convex-hull volume, surface area, area, and or perimeter. |
| Vertex Analysis | Describes the overall structure of a branched object based on topological and metrical properties. Root (or origin) point: For astrocytes, the origin is the point at which the structure is attached to the soma. Main types of vertices: V_d_ (bifurcation) or V_t_ (trifurcation): Nodal (or branching) points. V_p_: Terminal (or pendant) vertices. V_a_: primary vertices connecting 2 pendant vertices; V_b_: secondary vertices connecting 1 pendant vertex (V_p_) to 1 bifurcation (V_d_) or 1 trifurcation (V_t_); V_c_: tertiary vertices connecting either 2 bifurcations (V_d_), 2 trifurcations (V_t_), or 1 bifurcation (V_d_) and 1 trifurcation (V_t_). In the present report, we measured the number of vertices Va, Vb and Vc. |
| Complexity | Complexity = [Sum of the terminal orders + Number of terminals] × [Total branch length / Number of primary branches] |

**Supplementary Table 3.** Descriptive and comparative analysis, between young and aged mice, of reconstructed astrocytes in the dorsal region of the lacunosum-molecular stratum in CA1 under the HD diet.

|  | Descriptive Analysis - Dorsal Region - HD | | | | | |  | Comparative Analysis | | |
| --- | --- | --- | --- | --- | --- | --- | --- | --- | --- | --- |
|  | Mean |  | Median |  | Standard deviation |  |  |  | Student's t or Mann-Whitney U | |
|  |  |  |  |  |  |  |  |  |  |  |
| AGE | 6M | 18M | 6M | 18M | 6M | 18M |  |  |  |  |
|  |  |  |  |  |  |  |  |  |  | P value |
| **Length Total (µm)** | 341 | 299 | 311 | 286 | 189 | 111 |  | Length Total (µm) |  | 0.547 |
|  |  |  |  |  |  |  |  |  |  |  |
| **Mean Branch Lenght *** | 4.1 | 2.86 | 4.03 | 2.82 | 1.03 | 0.551 |  | Mean Branch Lenght |  | < .001 |
|  |  |  |  |  |  |  |  |  |  |  |
| **Tortuosity** | 1.17 | 1.17 | 1.15 | 1.16 | 0.0758 | 0.0347 |  | Tortuosity |  | 0.284 |
|  |  |  |  |  |  |  |  |  |  |  |
| **Surface Area(µm²) *** | 9.29 | 4.84 | 9.36 | 4.57 | 2.95 | 1.59 |  | Surface Area(µm²) |  | < .001 |
|  |  |  |  |  |  |  |  |  |  |  |
| **Base Diameter(µm)** | 0.904 | 0.739 | 0.884 | 0.653 | 0.166 | 0.308 |  | Base Diameter(µm) |  | < .001 |
|  |  |  |  |  |  |  |  |  |  |  |
| **Branch Volume(µm³)** | 175 | 107 | 156 | 92.6 | 131 | 58 |  | Branch Volume(µm³) |  | 0.004 |
|  |  |  |  |  |  |  |  |  |  |  |
| **Number of segments** | 83.7 | 162 | 72 | 102 | 47.6 | 169 |  | Number of segments |  | 0.001 |
|  |  |  |  |  |  |  |  |  |  |  |
| **Segments/mm** | 257 | 470 | 248 | 355 | 69.9 | 326 |  | Segments/mm |  | < .001 |
|  |  |  |  |  |  |  |  |  |  |  |
| **Number of trees** | 5.97 | 4.71 | 6 | 4 | 1.94 | 1.29 |  | Number of trees |  | < .001 |
|  |  |  |  |  |  |  |  |  | *** |  |
| **Total tree surface (µm²)** | 765 | 496 | 682 | 430 | 449 | 204 |  | Total tree surface (µm²) |  | < .001 |
|  |  |  |  |  |  |  |  |  |  |  |
| **Complexity** | 16610 | 26727 | 10214 | 17436 | 19505 | 26297 |  | Complexity |  | 0.005 |
|  |  |  |  |  |  |  |  |  |  |  |
| **VA** | 15.2 | 22.3 | 13 | 17 | 6.85 | 17.1 |  | VA |  | 0.02 |
|  |  |  |  |  |  |  |  |  |  |  |
| **VB** | 11.5 | 21.5 | 11 | 15 | 6.92 | 20.2 |  | VB |  | 0.001 |
|  |  |  |  |  |  |  |  |  |  |  |
| **VC** | 7.77 | 14.9 | 7 | 12 | 5.08 | 13 |  | VC |  | < .001 |
|  |  |  |  |  |  |  |  |  |  |  |
| **Planar angle*** | 47.4 | 50.8 | 47.6 | 50.6 | 6.24 | 4.53 |  | Planar angle |  | 0.001 |
|  |  |  |  |  |  |  |  |  |  |  |
| **CH Volume (µm³)** | 12880 | 3212 | 3223 | 2715 | 61605 | 1963 |  | CH Volume (µm³) |  | 0.152 |
|  |  |  |  |  |  |  |  |  |  |  |
| **CH Surface (µm²)** | 2998 | 1572 | 1981 | 1583 | 5991 | 513 |  | CH Surface (µm²) |  | 0.002 |
|  |  |  |  |  |  |  |  |  |  |  |
| **CH Area (µm²)** | 1111 | 643 | 861 | 656 | 899 | 201 |  | CH Area (µm²) |  | < .001 |
|  |  |  |  |  |  |  |  |  |  |  |
| **CH Perimeter (µm)** | 126 | 99.2 | 114 | 99.2 | 52.3 | 16.6 |  | CH Perimeter (µm) |  | < .001 |
|  |  |  |  |  |  |  |  |  |  |  |
| **K-DIM** | 1.08 | 1.09 | 1.07 | 1.08 | 0.0471 | 0.0449 |  | K-DIM |  | 0.045 |
|  |  |  |  |  |  |  |  |  |  |  |

**Supplementary Table 4.** Descriptive and comparative analysis, between young and aged mice, of reconstructed astrocytes in the dorsal region of the lacunosum-molecular stratum in CA1 under the HD/SD diet.

|  | Descriptive Analysis - Dorsal Region - HD/SD | | | | | |  | Comparative Analysis | | |
| --- | --- | --- | --- | --- | --- | --- | --- | --- | --- | --- |
|  | Mean |  | Median |  | Standard deviation |  |  |  |  |  |
|  |  |  |  |  |  |  |  |  |  |  |
| **AGE** | 6M | 18M | 6M | 18M | 6M | 18M |  |  |  | p value |
|  |  |  |  |  |  |  |  |  |  |  |
| **Length Total (µm)** | 392 | 224 | 379 | 192 | 143 | 110 |  | **Length Total (µm)** |  | < .001 |
|  |  |  |  |  |  |  |  |  |  |  |
| **Mean Branch Lenght (µm)** | 2.36 | 3.37 | 2.31 | 3.15 | 0.471 | 0.876 |  | **Mean Branch Lenght (µm)** |  | < .001 |
|  |  |  |  |  |  |  |  |  |  |  |
| **Tortuosity** | 1.16 | 1.14 | 1.16 | 1.13 | 0.0328 | 0.0414 |  | **Tortuosity** |  | 0.005 |
|  |  |  |  |  |  |  |  |  |  |  |
| **Surface Area(µm²)** | 3.85 | 7.06 | 3.4 | 6.46 | 1.46 | 2.42 |  | **Surface Area(µm²)** |  | < .001 |
|  |  |  |  |  |  |  |  |  |  |  |
| **Base Diameter(µm)*** | 0.584 | 0.84 | 0.588 | 0.83 | 0.147 | 0.136 |  | **Base Diameter(µm)** |  | < .001 |
|  |  |  |  |  |  |  |  |  |  |  |
| **Branch Volume(µm³)** | 135 | 103 | 113 | 85.9 | 76.5 | 61.7 |  | **Branch Volume(µm³)** |  | 0.022 |
|  |  |  |  |  |  |  |  |  |  |  |
| **Number of segments** | 174 | 74.2 | 164 | 59.5 | 74.1 | 47.1 |  | **Number of segments** |  | < .001 |
|  |  |  |  |  |  |  |  |  |  |  |
| **Segments/mm** | 438 | 316 | 432 | 318 | 80.1 | 75.8 |  | **Segments/mm** |  | < .001 |
|  |  |  |  |  |  |  |  |  |  |  |
| **Number of trees** | 5.21 | 4.88 | 5 | 5 | 1.45 | 1.26 |  | **Number of trees** |  | 0.26 |
|  |  |  |  |  |  |  |  |  |  |  |
| **Total tree surface (µm²)** | 602 | 462 | 560 | 433 | 266 | 233 |  | **Total tree surface (µm²)** |  | 0.004 |
|  |  |  |  |  |  |  |  |  |  |  |
| **Complexity** | 57778 | 12461 | 44231 | 7756 | 57998 | 15355 |  | **Complexity** |  | < .001 |
|  |  |  |  |  |  |  |  |  |  |  |
| **VA** | 28 | 12.9 | 27 | 11 | 10.8 | 7.31 |  | **VA** |  | < .001 |
|  |  |  |  |  |  |  |  |  |  |  |
| **VB** | 28 | 10.3 | 27 | 8 | 15 | 7.13 |  | **VB** |  | < .001 |
|  |  |  |  |  |  |  |  |  |  |  |
| **VC** | 20.7 | 7.47 | 19 | 5.5 | 9.39 | 6.11 |  | **VC** |  | < .001 |
|  |  |  |  |  |  |  |  |  |  |  |
| **Planar angle*** | 54 | 47.7 | 54.3 | 47.7 | 5.1 | 5.97 |  | **Planar angle** |  | < .001 |
|  |  |  |  |  |  |  |  |  |  |  |
| **CH Area (µm²)** | 755 | 4098 | 742 | 540 | 305 | 17244 |  | **CH Area (µm²)** |  | 0.04 |
|  |  |  |  |  |  |  |  |  |  |  |
| **CH Perimeter (µm)** | 106 | 108 | 104 | 94.1 | 20.9 | 71.6 |  | **CH Perimeter (µm)** |  | 0.028 |
|  |  |  |  |  |  |  |  |  |  |  |
| **CH Volume (µm³)** | 3728 | 2541 | 3275 | 1394 | 2316 | 3731 |  | **CH Volume (µm³)** |  | < .001 |
|  |  |  |  |  |  |  |  |  |  |  |
| **CH Surface (µm²)** | 1812 | 2248 | 1741 | 1147 | 651 | 5333 |  | **CH Surface (µm²)** |  | < .001 |
|  |  |  |  |  |  |  |  |  |  |  |
| **K-DIM** | 1.13 | 1.05 | 1.12 | 1.05 | 0.0535 | 0.0386 |  | **K-DIM** |  | < .001 |
|  |  |  |  |  |  |  |  |  |  |  |

**Supplementary Table 5.** Descriptive and comparative analysis, between young and aged mice, of reconstructed astrocytes in the dorsal region of the lacunosum-molecular stratum in CA1 under the HD/SD/HD diet.

|  | Descriptive Analysis – Dorsal Region – HD/SD/HD Regim | | | | | |  | Comparative Analysis | | |
| --- | --- | --- | --- | --- | --- | --- | --- | --- | --- | --- |
|  | Mean |  | Median |  | Standard deviation |  |  |  | Student's t or Mann-Whitney U | |
|  |  |  |  |  |  |  |  |  |  |  |
| AGE | 6M | 18M | 6M | 18M | 6M | 18M |  |  |  | p value |
|  |  |  |  |  |  |  |  |  |  |  |
| **Length Total (µm)** | 342 | 251 | 316 | 212 | 141 | 117 |  | **Length Total (µm)** |  | < .001 |
|  |  |  |  |  |  |  |  |  |  |  |
| **Mean Branch Lenght (µm)** | 2.47 | 3.77 | 2.39 | 3.66 | 0.413 | 0.997 |  | **Mean Branch Lenght (µm)** |  | < .001 |
|  |  |  |  |  |  |  |  |  |  |  |
| **Tortuosity** | 1.15 | 1.17 | 1.15 | 1.16 | 0.0284 | 0.0562 |  | **Tortuosity** |  | 0.01 |
|  |  |  |  |  |  |  |  |  |  |  |
| **Surface Area(µm²)** | 3.93 | 7.89 | 3.69 | 7.68 | 1.02 | 2.68 |  | **Surface Area(µm²)** |  | < .001 |
|  |  |  |  |  |  |  |  |  |  |  |
| **Base Diameter(µm)** | 0.599 | 0.819 | 0.582 | 0.819 | 0.125 | 0.129 |  | **Base Diameter(µm)** |  | < .001 |
|  |  |  |  |  |  |  |  |  |  |  |
| **Branch Volume(µm³)** | 121 | 117 | 108 | 109 | 66.3 | 60.4 |  | **Branch Volume(µm³)** |  | 0.792 |
|  |  |  |  |  |  |  |  |  |  |  |
| **Number of segments** | 143 | 73.2 | 126 | 60 | 67.9 | 42.1 |  | **Number of segments** |  | < .001 |
|  |  |  |  |  |  |  |  |  |  |  |
| **Segments/mm*** | 416 | 284 | 418 | 274 | 64.8 | 74.9 |  | **Segments/mm*** |  | < .001 |
|  |  |  |  |  |  |  |  |  |  |  |
| **Number of trees** | 4.97 | 5.15 | 5 | 5 | 1.5 | 1.76 |  | **Number of trees** |  | 0.711 |
|  |  |  |  |  |  |  |  |  |  |  |
| **Total tree surface (µm²)** | 544 | 522 | 502 | 475 | 244 | 246 |  | **Total tree surface (µm²)** |  | 0.54 |
|  |  |  |  |  |  |  |  |  |  |  |
| **Complexity** | 42496 | 12134 | 27737 | 6000 | 45178 | 15571 |  | **Complexity** |  | < .001 |
|  |  |  |  |  |  |  |  |  |  |  |
| **VA** | 22.3 | 13.7 | 20 | 12 | 10.1 | 6.74 |  | **VA** |  | < .001 |
|  |  |  |  |  |  |  |  |  |  |  |
| **VB** | 24 | 9.44 | 22 | 6 | 12.8 | 6.77 |  | **VB** |  | < .001 |
|  |  |  |  |  |  |  |  |  |  |  |
| **VC** | 15.2 | 7.98 | 14 | 6 | 8.28 | 6 |  | **VC** |  | < .001 |
|  |  |  |  |  |  |  |  |  |  |  |
| **Planar angle *** | 53.4 | 49.6 | 53 | 48.7 | 4.07 | 5.24 |  | **Planar angle *** |  | < .001 |
|  |  |  |  |  |  |  |  |  |  |  |
| **CH Volume (µm³)** | 6198 | 3727 | 4356 | 2577 | 7592 | 3862 |  | **CH Volume (µm³)** |  | 0.004 |
|  |  |  |  |  |  |  |  |  |  |  |
| **CH Surface (µm²)** | 2229 | 1692 | 1888 | 1438 | 2389 | 1172 |  | **CH Surface (µm²)** |  | 0.004 |
|  |  |  |  |  |  |  |  |  |  |  |
| **CH Area (µm²)** | 833 | 699 | 689 | 610 | 1030 | 509 |  | **CH Area (µm²)** |  | 0.049 |
|  |  |  |  |  |  |  |  |  |  |  |
| **CH Perimeter (µm)** | 109 | 104 | 106 | 98.9 | 49.7 | 37 |  | **CH Perimeter (µm)** |  | 0.178 |
|  |  |  |  |  |  |  |  |  |  |  |
| **K-DIM** | 1.11 | 1.06 | 1.1 | 1.05 | 0.0414 | 0.0389 |  | **K-DIM** |  | < .001 |
|  |  |  |  |  |  |  |  |  |  |  |

**Supplementary Table 6.** Descriptive and comparative analysis, between young and aged mice, of reconstructed astrocytes in the ventral region of the lacunosum-molecular stratum in CA1 under the HD diet.

|  | Descriptive Analysis – Ventral Region - HD Regim | | | | | | | Comparative Analysis | |
| --- | --- | --- | --- | --- | --- | --- | --- | --- | --- |
|  | Mean |  | Median |  | Standard deviation |  |  | Student's t or Mann-Whitney U | |
|  |  |  |  |  |  |  |  |  |  |
| **AGE** | 6M | 18M | 6M | 18M | 6M | 18M |  |  | p value |
|  |  |  |  |  |  |  |  |  |  |
| **Length Total (µm)** | 286 | 244 | 236 | 217 | 178 | 99.7 |  | **Length Total (µm)** | 0.463 |
|  |  |  |  |  |  |  |  |  |  |
| **Length media (µm)** | 4.24 | 3.33 | 4.3 | 3.26 | 1.16 | 0.526 |  | **Length media (µm)** | < .001 |
|  |  |  |  |  |  |  |  |  |  |
| **Tortuosity** | 1.15 | 1.18 | 1.15 | 1.17 | 0.036 | 0.0437 |  | **Tortuosity** | < .001 |
|  |  |  |  |  |  |  |  |  |  |
| **Surface Area(µm²)** | 9.97 | 5.87 | 10.4 | 5.59 | 3.3 | 1.55 |  | **Surface Area(µm²)** | < .001 |
|  |  |  |  |  |  |  |  |  |  |
| **Base diameter*** | 0.933 | 0.711 | 0.946 | 0.716 | 0.186 | 0.125 |  | **Branch Volume (µm³)** | < .001 |
|  |  |  |  |  |  |  |  |  |  |
| **Branch Volume (µm³)** | 171 | 96.8 | 129 | 80.6 | 145 | 71 |  | **Base diameter** | < .001 |
|  |  |  |  |  |  |  |  |  |  |
| **No of Segments** | 70.4 | 74.9 | 59 | 68.5 | 41.2 | 34.9 |  | **No of Segments** | 0.182 |
|  |  |  |  |  |  |  |  |  |  |
| **Segments/mm** | 262 | 301 | 231 | 306 | 114 | 62.6 |  | **Segments/mm** | 0.007 |
|  |  |  |  |  |  |  |  |  |  |
| **No of trees** | 5.51 | 4.75 | 5 | 5 | 1.8 | 1.07 |  | **No of trees** | 0.004 |
|  |  |  |  |  |  |  |  |  |  |
| **Total tree Surface(µm²)** | 680 | 426 | 535 | 380 | 469 | 198 |  | **Total tree Surface(µm²)** | < .001 |
|  |  |  |  |  |  |  |  |  |  |
| **Complexity** | 13619 | 12937 | 6087 | 8944 | 17643 | 12241 |  | **Complexity** | 0.106 |
|  |  |  |  |  |  |  |  |  |  |
| **VA** | 12.6 | 13.4 | 11 | 12 | 7.02 | 5.67 |  | **VA** | 0.164 |
|  |  |  |  |  |  |  |  |  |  |
| **VB** | 9.68 | 10.9 | 8 | 10 | 6.37 | 6.17 |  | **VB** | 0.11 |
|  |  |  |  |  |  |  |  |  |  |
| **VC** | 6.39 | 8.12 | 4.5 | 7 | 5.34 | 4.91 |  | **VC** | 0.005 |
|  |  |  |  |  |  |  |  |  |  |
| **Planar angle*** | 47.4 | 49.5 | 47.6 | 49.5 | 5.99 | 5.17 |  | **Planar angle*** | 0.014 |
|  |  |  |  |  |  |  |  |  |  |
| **CH Volume (µm³)** | 4385 | 8282 | 2993 | 2178 | 5347 | 28428 |  | **CH Volume (µm³)** | 0.028 |
|  |  |  |  |  |  |  |  |  |  |
| **CH Surface (µm²)** | 2040 | 2511 | 1696 | 1300 | 1315 | 5166 |  | **CH Surface (µm²)** | 0.009 |
|  |  |  |  |  |  |  |  |  |  |
| **CH Area (µm²)** | 865 | 1121 | 744 | 526 | 552 | 2489 |  | **CH Area (µm²)** | 0.012 |
|  |  |  |  |  |  |  |  |  |  |
| **CH Perimeter (µm)** | 119 | 129 | 107 | 93.7 | 67.9 | 159 |  | **CH Perimeter (µm)** | 0.003 |
|  |  |  |  |  |  |  |  |  |  |
| **KDIM** | 1.06 | 1.07 | 1.05 | 1.07 | 0.0473 | 0.0433 |  | **KDIM** | 0.016 |
|  |  |  |  |  |  |  |  |  |  |

**Supplementary Table 7.** Descriptive and comparative analysis, between young and aged mice, of reconstructed astrocytes in the ventral region of the lacunosum-molecular stratum in CA1 under the HD/SD diet.

|  | Descriptive Analysis – Ventral Region – HD/SD Regim | | | | | |  |  | Comparative Analysis | |
| --- | --- | --- | --- | --- | --- | --- | --- | --- | --- | --- |
|  | Mean |  | Median |  | Standard deviation |  |  |  | Student's t or Mann-Whitney U | |
|  |  |  |  |  |  |  |  |  |  |  |
| **AGE** | 6M | 18M | 6M | 18M | 6M | 18M |  |  |  |  |
|  |  |  |  |  |  |  |  |  |  | p value |
| **Length Total (µm)** | **414** | **237** | 376 | 228 | 179 | 109 |  | **Length Total (µm)** |  | < .001 |
|  |  |  |  |  |  |  |  |  |  |  |
| **Length media (µm)** | 2.18 | 3.41 | 2.13 | 3.38 | 0.349 | 0.799 |  | **Length media (µm)** |  | < .001 |
|  |  |  |  |  |  |  |  |  |  |  |
| **Tortuosity** | 1.18 | 1.14 | 1.15 | 1.13 | 0.151 | 0.0316 |  | **Tortuosity** |  | 0.001 |
|  |  |  |  |  |  |  |  |  |  |  |
| **Surface Area(µm²)** | 3.34 | 7.03 | 3.23 | 6.92 | 0.956 | 2.02 |  | **Surface Area(µm²)** |  | < .001 |
|  |  |  |  |  |  |  |  |  |  |  |
| **Base diameter*** | 0.56 | 0.806 | 0.555 | 0.81 | 0.0978 | 0.125 |  | **Base diameter** |  | < .001 |
|  |  |  |  |  |  |  |  |  |  |  |
| **Branch Volume (µm³)** | 137 | 110 | 111 | 104 | 78.8 | 65.3 |  | **Segment-Dendrites Volume** |  | 0.065 |
|  |  |  |  |  |  |  |  |  |  |  |
| **No of Segments** | 199 | 74.5 | 184 | 68.5 | 94.9 | 39.4 |  | **No of Segments** |  | 0.654 |
|  |  |  |  |  |  |  |  |  |  |  |
| **Segments/mm*** | 469 | 309 | 471 | 296 | 68.1 | 71 |  | **Segments/mm** |  | 0.787 |
|  |  |  |  |  |  |  |  |  |  |  |
| **No of trees** | 5.2 | 5.14 | 5 | 5 | 1.55 | 1.34 |  | **No of trees** |  | 0.654 |
|  |  |  |  |  |  |  |  |  |  |  |
| **Total tree surface(µm²)** | 623 | 489 | 549 | 465 | 307 | 245 |  | **Total tree surface(µm²)** |  | 0.005 |
|  |  |  |  |  |  |  |  |  |  |  |
| **Complexity** | 85000 | 11237 | 56732 | 8174 | 94383 | 11050 |  | **Complexity** |  | < .001 |
|  |  |  |  |  |  |  |  |  |  |  |
| **VA** | 31.2 | 13.7 | 28 | 13 | 13 | 6.57 |  | **VA** |  | < .001 |
|  |  |  |  |  |  |  |  |  |  |  |
| **VB** | 31.1 | 10.3 | 26.5 | 10 | 17.2 | 6.76 |  | **VB** |  | < .001 |
|  |  |  |  |  |  |  |  |  |  |  |
| **VC** | 23.6 | 7.66 | 21 | 7 | 11.8 | 5.25 |  | **VC** |  | < .001 |
|  |  |  |  |  |  |  |  |  |  |  |
| **Planar angle** | 54.6 | 46 | 55 | 46 | 4.68 | 5.35 |  | **Planar angle** |  | < .001 |
|  |  |  |  |  |  |  |  |  |  |  |
| **CH Volume (µm³)** | 3885 | 3201 | 2795 | 2073 | 3138 | 5333 |  | **CH Volume (µm³)** |  | 0.003 |
|  |  |  |  |  |  |  |  |  |  |  |
| **CH Surface (µm²)** | 1725 | 1725 | 1547 | 1437 | 704 | 1989 |  | **CH Surface (µm²)** |  | 0.068 |
|  |  |  |  |  |  |  |  |  |  |  |
| **CH Area (µm²)** | 701 | 787 | 671 | 653 | 261 | 954 |  | **CH Area (µm²)** |  | 0.594 |
|  |  |  |  |  |  |  |  |  |  |  |
| **CH Perimeter (µm)** | 101 | 107 | 100 | 102 | 18.2 | 55.3 |  | **CH Perimeter (µm)** |  | 0.98 |
|  |  |  |  |  |  |  |  |  |  |  |
| **K-DIM** | 1.14 | 1.06 | 1.15 | 1.06 | 0.0516 | 0.0399 |  | **KDIM** |  | < .001 |
|  |  |  |  |  |  |  |  |  |  |  |

**Supplementary Table 8.** Descriptive and comparative analysis, between young and aged mice, of reconstructed astrocytes in the ventral region of the lacunosum-molecular stratum in CA1 under the HD/SD/HD diet.

|  | Descriptive Analysis - Ventral Region - HD/SD/HD Regim | | | | | |  |  | Comparative Analysis | |
| --- | --- | --- | --- | --- | --- | --- | --- | --- | --- | --- |
|  | Mean |  | Median |  | Standard deviation |  |  |  | Student's t or Mann-Whitney U | |
|  |  |  |  |  |  |  |  |  |  |  |
| **AGE** | 6M | 18M | 6M | 18M | 6M | 18M |  |  |  |  |
|  |  |  |  |  |  |  |  |  |  | p value |
| **Length Total (µm)** | 329 | 253 | 310 | 222 | 137 | 122 |  | **Length Total (µm)** |  | < .001 |
|  |  |  |  |  |  |  |  |  |  |  |
| **Length media (µm)*** | 2.53 | 4 | 2.45 | 4.13 | 0.394 | 0.917 |  | **Length media (µm)*** |  | < .001 |
|  |  |  |  |  |  |  |  |  |  |  |
| **Tortuosity** | 1.15 | 1.16 | 1.14 | 1.15 | 0.0387 | 0.0491 |  | **Tortuosity** |  | 0.061 |
|  |  |  |  |  |  |  |  |  |  |  |
| **Surface Area(µm²)** | 4.15 | 8.81 | 3.95 | 8.51 | 0.986 | 2.9 |  | **Surface Area(µm²)** |  | < .001 |
|  |  |  |  |  |  |  |  |  |  |  |
| **Base diameter*** | 0.611 | 0.872 | 0.616 | 0.875 | 0.0964 | 0.167 |  | **Base diameter*** |  | < .001 |
|  |  |  |  |  |  |  |  |  |  |  |
| **Branch Volume (µm³)** | 115 | 128 | 102 | 106 | 54.9 | 82 |  | **Branch Volume (µm³)** |  | 0.79 |
|  |  |  |  |  |  |  |  |  |  |  |
| **No of Segments** | 137 | 69.8 | 125 | 55 | 69.3 | 41.4 |  | **No of Segments** |  | < .001 |
|  |  |  |  |  |  |  |  |  |  |  |
| **Segments/mm** | 405 | 265 | 408 | 242 | 61.4 | 68.5 |  | **Segments/mm** |  | < .001 |
|  |  |  |  |  |  |  |  |  |  |  |
| **No of trees** | 4.93 | 4.81 | 5 | 5 | 1.3 | 1.38 |  | **No of trees** |  | 0.584 |
|  |  |  |  |  |  |  |  |  |  |  |
| **Total tree surface(µm²)** | 535 | 544 | 483 | 485 | 227 | 275 |  | **Total tree surface(µm²)** |  | 0.834 |
|  |  |  |  |  |  |  |  |  |  |  |
| **Complexity** | 39610 | 12007 | 23769 | 6652 | 47537 | 12163 |  | **Complexity** |  | < .001 |
|  |  |  |  |  |  |  |  |  |  |  |
| **VA** | 21.4 | 12.7 | 18 | 10 | 10.4 | 6.98 |  | **VA** |  | < .001 |
|  |  |  |  |  |  |  |  |  |  |  |
| **VB** | 20.2 | 9.71 | 19 | 8 | 12 | 6.58 |  | **VB** |  | < .001 |
|  |  |  |  |  |  |  |  |  |  |  |
| **VC** | 14.7 | 7.33 | 13 | 5 | 8.79 | 5.56 |  | **VC** |  | < .001 |
|  |  |  |  |  |  |  |  |  |  |  |
| **Planar angle** | 53.5 | 48.4 | 53.8 | 48.3 | 3.3 | 6.87 |  | **Planar angle** |  | < .001 |
|  |  |  |  |  |  |  |  |  |  |  |
| **CH Volume (µm³)** | 4399 | 3409 | 2579 | 2443 | 10858 | 2805 |  | **CH Volume (µm³)** |  | 0.618 |
|  |  |  |  |  |  |  |  |  |  |  |
| **CH Surface (µm²)** | 1587 | 1660 | 1441 | 1559 | 641 | 603 |  | **CH Surface (µm²)** |  | 0.462 |
|  |  |  |  |  |  |  |  |  |  |  |
| **CH Area (µm²)*** | 675 | 694 | 620 | 676 | 259 | 224 |  | **CH Area (µm²)*** |  | 0.648 |
|  |  |  |  |  |  |  |  |  |  |  |
| **CH Perimeter (µm)** | 99.8 | 103 | 96 | 104 | 20.1 | 17.3 |  | **CH Perimeter (µm)** |  | 0.356 |
|  |  |  |  |  |  |  |  |  |  |  |
| **KDIM** | 1.1 | 1.06 | 1.1 | 1.05 | 0.0484 | 0.0482 |  | **KDIM** |  | < .001 |
|  |  |  |  |  |  |  |  |  |  |  |

**Supplementary Table 9. Comparative analysis between low-complexity astrocytes (AST2) from young (6M) and aged (18M) murine mice using two-way ANOVA and Cohen's D effect size.**

| COMPLEXIDADE | | | | | | | | | | |
| --- | --- | --- | --- | --- | --- | --- | --- | --- | --- | --- |
| AST2 DORSAL | | | | | | | | | | |
| Source of variations | F | p |  | **Effect Size – Cohen's d** |  |  |  |  |  |  |
| Diets | 19.9 | < .001 |  |  |  |  |  |  |  |  |
| Age x Diet | 53.8 | < .001 |  | HD 6M vs HD 18M | 0.999 |  |  |  |  |  |
|  |  |  |  | HD/SD 6M vs HD/SD 18M | -1.73 |  |  |  |  |  |
|  |  |  |  | HD/SD/SD 6M vs HD/SD/HD 18M | -1.56 |  |  |  |  |  |
| Descriptives |  |  |  | **Comparisions** |  |  |  |  |  |  |
| Diets | Age | Mean | SD | **Diets** |  |  | **Regions** |  |  |  |
| HD | 6M | 4980 | 3637 | HD 6M | HD/SD 6M | < .001 | HD 6M | HD 18M | 0.011 |  |
|  | 18M | 22735 | 21324 | HD 6M | HD/SD/HD 6M | 0.004 | HDSD 6M | HDSD 18M | < .001 |  |
| HD/SD | 6M | 65078 | 45249 | HD/SD 6M | HD/SD/HD 6M | < .001 | HDSDHD 6M | HDSDHD 18M | < .001 |  |
|  | 18M | 4093 | 2666 | HD 18M | HD/SD 18M | 0.005 |  |  |  |  |
| HD/SD/HD | 6M | 23608 | 13892 | HD 18M | HD/SD/HD 18M | 0.002 |  |  |  |  |
|  | 18M | 6542 | 4694 | HD/SD 18M | HD/SD/HD 18M | 0.997 |  |  |  |  |

**Supplementary Table 10. Comparative analysis between high-complexity astrocytes (AST1) from young (6M) and aged (18M) murine mice using two-way ANOVA and Cohen's D effect size.**

| COMPLEXITY | | | | | | | | | |
| --- | --- | --- | --- | --- | --- | --- | --- | --- | --- |
| AST1 DORSAL | | | | | | | | | |
| Source of variations | F | p |  | **Effect Size – Cohen's d** |  |  |  |  |  |
| Diets | 0.618 | 0.541 |  |  |  |  |  |  |  |
| Age x Diet | 12.414 | < .001 |  | HD 6M vs HD 18M | 2.73 |  |  |  |  |
|  |  |  |  | HD/SD 6M vs HD/SD 18M | -1.31 |  |  |  |  |
|  |  |  |  | HD/SD/SD 6M vs HD/SD/HD 18M | -1.27 |  |  |  |  |
| Descriptives |  |  |  | **Comparisions** |  |  |  |  |  |
| Diets | Age | Mean | SD | **Diets** |  |  | **Regions** |  |  |
| HD | 6M | 24364 | 21865 | HD 6M | HD/SD 6M | < .001 | HD 6M | HD 18M | 0.116 |
|  | 18M | 83417 | 19092 | HD 6M | HD/SD/HD 6M | < .001 | HDSD 6M | HDSD 18M | < .001 |
| HD/SD | 6M | 96652 | 91074 | HD/SD 6M | HD/SD/HD 6M | 1 | HDSDHD 6M | HDSDHD 18M | 0.003 |
|  | 18M | 24302 | 17030 | HD 18M | HD/SD 18M | 0.133 |  |  |  |
| HD/SD/HD | 6M | 100018 | 57809 | HD 18M | HD/SD/HD 18M | 0.473 |  |  |  |
|  | 18M | 37552 | 21903 | HD/SD 18M | HD/SD/HD 18M | 0.961 |  |  |  |
